# Analysis of a new begomovirus unveils a composite element conserved in the *CP* gene promoters of several *Geminiviridae* genera: Clues to comprehend the complex regulation of late genes

**DOI:** 10.1371/journal.pone.0210485

**Published:** 2019-01-23

**Authors:** Mariana Cantú-Iris, Guillermo Pastor-Palacios, Jorge Armando Mauricio-Castillo, Bernardo Bañuelos-Hernández, Jesús Aarón Avalos-Calleros, Alejandro Juárez-Reyes, Rafael Rivera-Bustamante, Gerardo R. Argüello-Astorga

**Affiliations:** 1 División de Biología Molecular, Instituto Potosino de Investigación Científica y Tecnológica A. C., San Luis Potosí, SLP, México; 2 CONACYT–CIIDZA–Instituto Potosino de Investigación Científica y Tecnológica A. C., San Luis Potosí, SLP, México; 3 Unidad Académica de Agronomía, Universidad Autónoma de Zacatecas, Cieneguillas, Zacatecas, México; 4 Facultad de Agronomía y Veterinaria, Universidad De La Salle Bajio, Avenida Universidad 602, Lomas del campestre, León Guanajuato, México; 5 Departamento de Ingeniería Genética de Plantas, Centro de Investigación y de Estudios Avanzados del IPN, Irapuato, Gto., México; Washington State University, UNITED STATES

## Abstract

A novel bipartite begomovirus, *Blechum interveinal chlorosis virus* (BleICV), was characterized at the genome level. Comparative analyses revealed that BleICV coat protein (*CP*) gene promoter is highly divergent from the equivalent region of other begomoviruses (BGVs), with the single exception of *Tomato chino La Paz virus* (ToChLPV) with which it shares a 23-bp phylogenetic footprint exhibiting dyad symmetry. Systematic examination of the homologous *CP* promoter segment of 132 New World BGVs revealed the existence of a quasi-palindromic DNA segment displaying a strongly conserved ACTT-(N7)-AAGT core. The spacer sequence between the palindromic motifs is constant in length, but its sequence is highly variable among viral species, presenting a relaxed consensus (TT)GGKCCCY, which is similar to the Conserved Late Element or CLE (GTGGTCCC), a putative TrAP-responsive element. The homologous *CP* promoter region of Old World BGVs exhibited a distinct organization, with the putative TATA-box overlapping the left half of the ACTT-N7 composite element. Similar *CP* promoter sequences, dubbed “TATA-associated composite element” or TACE, were found in viruses belonging to different *Geminiviridae* genera, hence hinting unsuspected evolutionary relationships among those lineages. To get cues about the TACE function, the regulatory function of the CLE was explored in distinct experimental systems. Transgenic tobacco plants harboring a *GUS* reporter gene driven by a promoter composed by CLE multimers expressed high beta-glucuronidase activity in absence of viral factors, and that expression was increased by begomovirus infection. On the other hand, the TrAP-responsiveness of a truncated *CP* promoter of *Tomato golden mosaic virus* (TGMV) was abolished by site-directed mutation of the only CLE present in it, whereas the artificial addition of one CLE to the -125 truncated promoter strongly enhanced the transactivation level in tobacco protoplasts. These results indicate that the CLE is a TrAP-responsive element, hence providing valuable clues to interpret the recurrent association of the CLE with the TACE. On the basis of the aforesaid direct evidences and the insights afforded by the extensive comparative analysis of BleICV *CP* promoter, we propose that the TACE might be involved in the TrAP-mediated derepression of *CP* gene in vascular tissues.

## Introduction

The family *Geminiviridae* is the largest group of plant viruses, with >440 recognized species distributed throughout all land ecosystems with warm and temperate climates around the world [1]. Geminiviruses have small genomes composed of one or two circular single-stranded DNA molecules encapsulated into twinned quasi-icosahedral virions [2]. These viruses infect a broad variety of wild plants and agricultural crops, causing significant shrinkage of staple food supplies and huge economic losses worldwide [3,4]. Geminiviruses are transmitted by sap-sucking insects in the order Hemiptera [5], and the transmission process is highly specific and dependent on the viral coat protein (CP) [6,7]. Accordingly, the insect vector and the virus genome organization are the main criteria to classify the family *Geminiviridae* into separate taxons. Nine genera are currently recognized: *Becurtovirus*, *Begomovirus*, *Capulavirus*, *Curtovirus*, *Eragrovirus*, *Grablovirus*, *Mastrevirus*, *Topocuvirus* and *Turncurtovirus* [8,9].The begomoviruses (BGVs) constitute the largest genus, comprising nearly 88% of all geminivirus species (https://talk.ictvonline.org/taxonomy/). BGVs solely infect dicots, and are transmitted by the cosmopolitan agricultural pest *Bemisia tabaci*, a complex of cryptic, morphologically indistinguishable whitefly species [10,11]. Two major lineages of BGVs have been largely recognized on the basis of their genome arrangement and geographical distribution: the Old World (OW: Eurasia, Africa, and Oceania) and the New World BGVs (NW: the Americas) [12]. With only two reported exceptions [13,14] the NW BGVs possess a bipartite genome (DNA-A and DNA-B), whereas the OW BGVs include both bipartite and monopartite species.

The DNA-A of NW BGVs typically contains five open reading frames (ORFs), one in the virion-sense strand (*AV1* or *CP*) encoding the coat protein, and four in the complementary sense (*AC1* or *Rep*; *AC2* or *Trap*; *AC3* or *Ren;* and *AC4*) encoding proteins involved in a variety of essential functions for the virus infective cycle: replication, interference of the plant cell cycle, temporal regulation of viral gene expression, and suppression of host antiviral responses [15,16]. The DNA-B, encodes two proteins, BV1 or NSP, and BC1 or MP, which participate in the intra- and intercellular movement of viral DNA, respectively [15,16]. The DNA-A and DNA-B molecules of bipartite BGVs are very different in sequence, with the exception of a shared DNA segment ranging from 140 to 200-nt in length, which contains the virus replication origin. The *Ori*, also known as “common region” in bipartite BGVs, is a part of the intergenic region (IR) that includes bidirectional promoters controlling the expression of the virion-sense and the complementary-sense genes of each genomic component.

The *CP* gene is expressed at the late stage of the infection process, and is regulated at the transcriptional level by a small (~14 kDa) multifunctional protein termed “transcriptional activator protein” (TrAP), encoded by the *AC2*/*C2*/*Trap* gene [16]. TrAP is expressed at the early stage of the infection process and subsequently activates the expression of the late genes (i.e., *CP* and the two genes encoded in the DNA-B) [17–20]. The transactivator has three discernable domains: a basic N-terminal domain including a nuclear localization signal, a central region that contains an atypical zinc-finger motif, and a C-terminal acidic activation domain that is critical for its function as transcriptional activator [21]. The last function is not virus specific as it has been demonstrated in studies of complementation of *AC2* mutants [22, 23] and transactivation of heterologous *CP* promoters fused to a reporter gene [24]. Moreover, TrAP does not bind dsDNA in a sequence-specific manner [25, 26], like other well-characterized viral transactivators (e,g., herpesvirus VP16 and adenovirus E1A) that recognize their target elements in viral promoters through interaction with host transcription factors that do bind specific DNA sequences [27].

Diverse studies with transgenic plants harboring chimeric genes with the begomovirus *CP* promoter fused to the *GUS* reporter gene, have shown that the regulation of *CP* gene expression is a complex process interweaving the action of different kinds of regulatory elements: i) a transcriptional silencer which suppresses the activity of the *CP* promoter in vascular tissues in absence of TrAP; this silencer was mapped in *Tomato golden mosaic virus* (TGMV) into a ~300 bp segment encompassing a part of the *AC2*/*Trap* and *AC3/Ren* genes [28], and ii) one or more *cis*-acting positive elements that activate the *CP* promoter in mesophyll cells of infected plants [28]. Lacatus and Sunter [29] demonstrated that the sequences mediating repression and activation of TGMV and *Cabbage leaf curl virus* (CbLCuV) *CP* promoters are bound by different nuclear factors common to three plant species, whereas TGMV TrAP can interact with both sequences independently, as it was determined by chromatin immunoprecipitation assays. These observations indicate that TrAP could interact with diverse plant transcription factors, in addition to the *Arabidopsis* PEAPOD-2 protein [30] to differentially regulate the *CP* promoter activity in mesophyll and vascular tissues. The former evidence and the lack of virus-specific functions of TrAP naturally lead to the idea that promoters of BGV late genes might contain shared sequences functioning as TrAP-responsive elements.

In an early comparative analysis of the intergenic region of bipartite and monopartite BGVs, a sequence motif (i.e., GTGGTCCC) was identified in many *CP* and *BV1* (*NSP*) promoters. This sequence, termed "conserved late element” (CLE), was postulated to be a functional target for TrAP [31]. A subsequent study of the *Pepper huasteco yellow vein virus* (PHYVV) *CP* promoter lent experimental support to the CLE hypothesis. Transgenic tobacco plants with a truncated *CP* promoter, termed -115*CP*, fused to the *GUS* gene exhibited barely detectable glucuronidase activity in all analyzed tissues; this activity was strongly increased with infection by PHYVV, hence indicating that the truncated -115*CP* promoter is still responsive to the viral transactivator. This short promoter contains three sequences identical to the CLE [32]. Furthermore, synthetic promoters including one or two CLEs fused to the CaMV 35S minimal promoter exhibited TrAP-responsiveness in transient expression assays [32]. The actual importance of the CLE in the transactivation of TGMV *CP* promoter was questioned in a study by Sunter and Bisaro [33] who deleted the only canonical CLE present in a -657 *CP* promoter:*GUS* construct and did not observe significant effect on the TrAP-responsiveness of the *CP* promoter. However, in the same study the authors delimited the minimal TGMV *CP* promoter which is responsive to the transactivator, and concluded that an element essential for TrAP-mediated activation lies between -125 and -107 (5´-CGTCTAAGTGGTCCCGCA-3’), an 18-bp long region where the CLE is located [33]. Since the latter element is presumably irrelevant for the transactivation process, and the sequences flanking the CLE in the -125 and -107 region are not conserved in other BGVs, the conclusions of this study seem paradoxical, and deserve a careful analysis. More recent advances in the knowledge of the complex regulation of late promoters of BGVs and other geminiviruses were reviewed by Borah et al. [34].

In this work, we describe a new begomovirus which possesses a *CP* promoter with an atypical assortment of putative *cis*-regulatory elements. Comparative analyses of that *CP* promoter led to discover a conserved complex element with partial dyad symmetry, which is closely associated to the TATA box.

## Materials and methods

*Blechum piramidatum* (Acanthaceae) plants with symptoms of interveinal chlorosis were found in an area of wild vegetation located between the town of Nohacal and the archaeological site of Edzna (19° 36´14.78´´N; 90° 19´ 10.44´´W), in Campeche, Mexico. This area is outside the protected area of Edzna, and no specific permission is required to collect.

### Virus source and cloning of genomic components

Leaf material from four symptomatic plants of the weed *B*.*pyramidatum* were collected (November 2011) in an area of wild vegetation site between the town Nohacal and the archaeological zone of Edzná, in Campeche, Mexico. Total DNA was obtained from dried leaf samples by a modified Dellaporta method [35]. To increase both the quantity and quality of the DNA for subsequent manipulations, the extracts were subjected to rolling circle amplification (RCA) by using the TempliPhi kit (New England Biolabs) following the recommendations of the manufacturer. The existence of BGVs in the samples was assessed by PCR using several degenerate primers: RepDGR-for/ CpYMAC-rev [36], RepYIDK-rev (5´-CAAGTCCTACATCGACAAGGAYGGAGA-3) and Cp-EGP70-for (5´-GGTTGTGAAGGNCCNTGTAAGGTYCA-3´). The DNA-A was amplified by PCR using two pairs of primers that produce amplicons overlapping along a ~520-bp segment, and that jointly encompass the full A genomic component. The DNA-B was PCR amplified using two sets of degenerate primers, BC1-290rev/BV1-310for and BC1-290for/BV1-470-rev, as previously described [37]; the latter primers are complementary to conserved sequences in the B genomic component of New World BGVs. The amplicons were cloned into pGEM-T Easy Vector (Promega) and analysed in restriction fragment length polymorphism (RFLP) assays using *EcoRI* and *HinfI* endonucleases. The digestion products were separated by electrophoresis in 2.5% agarose gels, and PCR clones exhibiting distinct restriction patterns were identified. Independent clones with a similar restriction pattern were sequenced and full-length contigs were then assembled.

### DNA sequencing and sequence analysis

Automated sequencing was carried out at LANBAMA (IPICYT, San Luis Potosí, Mexico) using a 3130 Genetic Analyzer (Applied Biosystems). The overlapping DNA fragments were subsequently assembled using SeqMan of DNAStar software (DNAStar Inc., Madison, WI, USA). The assembled sequences of the DNA-A and DNA-B components were compared with those from the NCBI database using the Nucleotide Basic Local Alignment Search Tool (BLASTn). Sequences with the highest scores were selected for comparison by alignment using MUSCLE [38] and pairwise nucleotide sequence identities were calculated with Sequence Demarcation Tool (SDT v1.2) [39].

### Phylogenetic analyses

For phylogenetic analyses of DNA-A and DNA-B of BleICV and representative BGVs, sequences were aligned in MEGA 7 software using Clustal W [40]. Phylogenetic trees were constructed using maximum-likelihood (ML) method based on the Tamura-Nei model. The tree support was tested by bootstrapping with 1000 replicates. For determining the percent nucleotide identity, viral sequences were aligned by MUSCLE in the sequence demarcation tool version 1.2 (SDTv1.2) software. The GenBank accession numbers of BGVs used in DNA-A and DNA-B sequence comparisons and phylogenetic reconstruction are listed in S1 Table.

### Phylogenetic-structural analysis of non-coding sequences

Since conventional computer programs for DNA sequence comparisons usually fail to detect evolutionarily related but structurally variable promoter regions, several alternative approaches, such as the “phylogenetic-structural method” of sequence analysis [41], have been devised. This method is based on the search of “homologous” (rather than only “similar”) non-coding DNA sequences. In this study we identified homologous putative *cis*-regulatory elements by looking for conserved arrays of specific DNA motifs in geminivirus *CP* promoters. This was accomplished both by computer-assisted searches and/or by visual inspection of equivalent promoter segments. The criteria to identify the putative TATA box of the CP gene were its similarity to the TATA box consensus (i.e., TATAWWW), its position and distance relative to the start codon of the *CP* gene (in NW BGVs) or, in the case of geminiviruses with overlapped genes in the virion sense (e.g., OW BGVs, curtoviruses and topocuviruses), the TA-rich sequence closest to the first ORF in the set of genes overlapping the *CP* gene.

### Construction of chimeric promoters with CLE multimers

To construct integrative vectors pBI46S-3CLE, pBI90S-3CLE and pBI46S-6CLE which contain multiple copies of the CLE element in the same orientation, a synthetic dsDNA fragment containing 3 copies of the CLE with 8bp spacing and *HindIII* and *SpeI* restriction sites located at the ends was designed (S3 Table). The oligonucleotides were resuspended at 100ng/μl in a buffer, containing Tris 20mM pH 7.5, MgCl_2_ 10mM and NaCl 250mM, heated to 85°C in a water bath for 5 minutes and were taken out to reach room temperature. The dsDNA fragment obtained was ligated into *HindIII -SpeI* sites upstream of truncated -46 and -90 CaMV 35S promoter of vectors pBI46S and pBI90S [42]. The truncated -46 promoter represents a minimal promoter which contains the TATA box and an initiator element. In contrast, truncated promoter -90 contains in addition an AS-1 transcriptional element. To generate pBI46S-6CLE an additional dsDNA fragment (B) *Hind III* compatible was cloned upstream the 3CLE copies of pBI46S-3CLE.

### Generation of tobacco transgenic plants

Selected leaves of *Nicotiana tabacum* plants were sliced into 0.5 x 0.5 cm squares and placed on solid MS medium in contact to a suspension of *Agrobacterium tumefaciens* harbouring the plasmids with the promoter constructs. The leaf fragments were transferred to a MS medium with growth regulators (2mg/l 6-benzylaminopurine, BAP), 150mg/l kanamycin, 300mg/l cefotaxime, to select transformed plants using two consecutive rounds of selection. In this media the kanamycin resistance marker which is linked to promoter construct cassettes selects for transformant plants. Plants were removed and placed in MS solid medium without growth regulators. The plants were maintained at 25°C and 16/8h light-dark cycles for the rest of the experiments.

### Constructs for the study of *Tomato golden mosaic virus* (TGMV) *CP* promoter

A series of progressively shorter versions of *CP* promoter of TGMV were generated by PCR amplification of *CP* promoter using designed primers (S3 Table) and cloned into pBSGUS cut with *HindIII* and *XbaI*. The generated truncated promoters were -184, -125 and -107. The pBSGUS is a pBlueScript KS-derived vector harbouring the reporter gene *uidA* (GUS) and an MCS for insert promoters. The constructs generated reproduced a series of promoter deletions used by Sunter and Bisaro [33] to characterize a minimal sequence required for CP gene activation in the same virus. As control, a pair of synthetic modified-promoter constructs were generated using the same methodology. The -125 CLEmut construct was generated by using an oligonucleotide which contained a mutated CLE (changing GTGGTCCC to GTAATAAC) at the native CLE position found on the -125 construct; the construct -125(2CLE) adds an extra copy of CLE upstream of the -125 promoter construct. The relevant primers are listed in S3 Table.

### Transient expression assays in NT1 suspension cell protoplasts

The different module-promoter constructs were tested by transient expression assays in protoplasts prepared from *Nicotiana tabacum* NT1 suspension cell cultures. Protoplasts were obtained from cells at the logarithmic growth phase after treatment with an enzymatic solution containing a mixture of *Trichoderma viride* cellulase and *Aspergillus japonicum* pectolyase (Sigma-Aldrich Co.). Protoplast transfection was performed by electroporation, using 15μg of purified DNA from expression vectors and 500μF and 250v pulses in a Bio-Rad Gene Pulser Xcell^TM^. Treated cells were then incubated at 25°C for 48hrs. Protoplasts were harvested and total protein was measured by the Bradford method. beta-glucuronidase activity was measured in a GENios TECAN fluorescence reader through the quantification of the coloured MU product of hydrolysis of MUG catalyzed by the product of the gene *uidA* (GUS) using wavelengths λ_ex_ = 355–372 nm and λ_em_ = 440–480 nm.

## Results

### Isolation and characterization of a novel begomovirus

**Fig 1:**
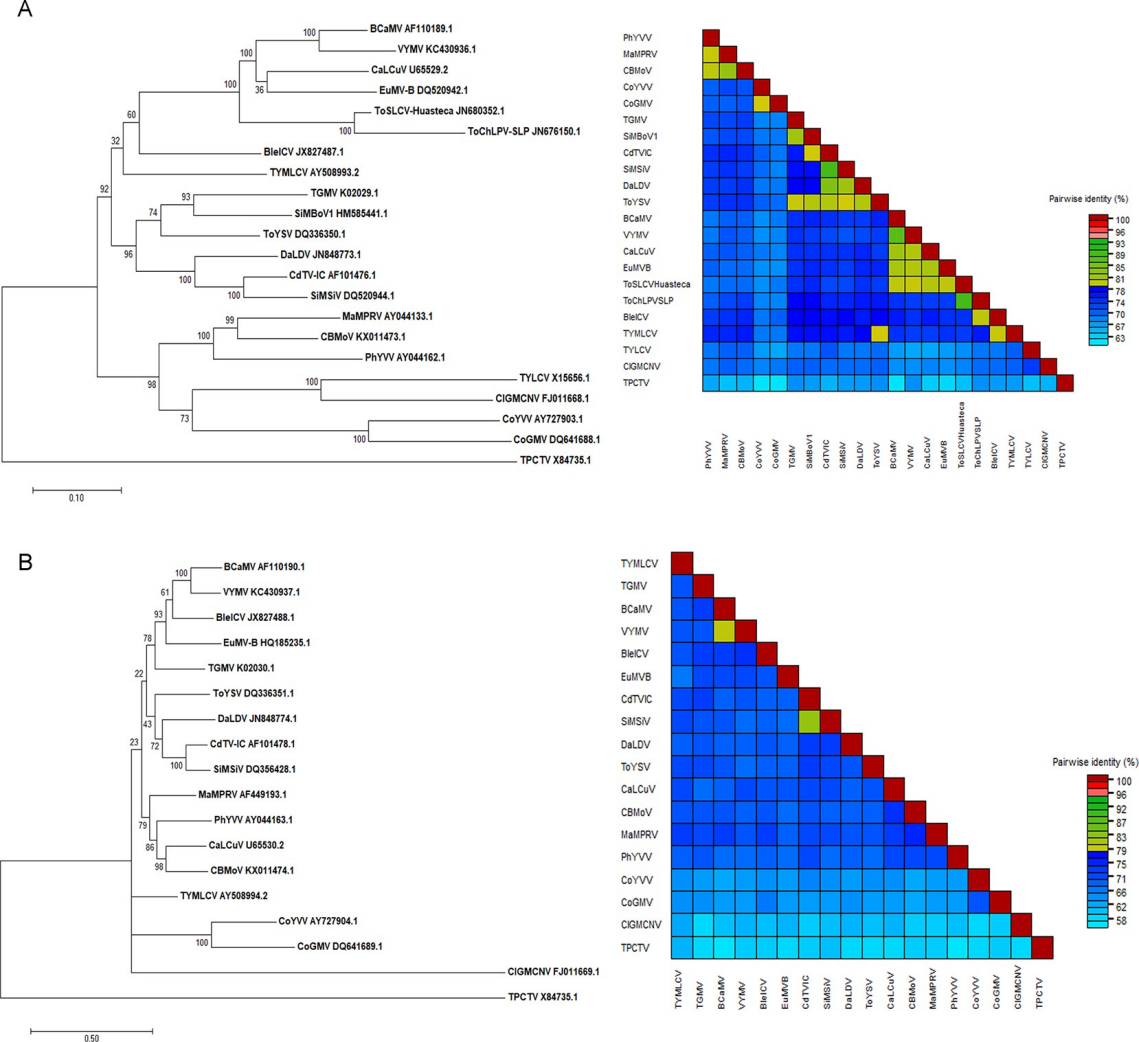
**Maximum likelihood (ML) phylogenetic tree and colour coded matrix of pairwise sequence identity based on full-length sequences of genome components A and B.** (A) DNA-A; (B) DNA-B. ML phylogenies were constructed by multiple alignments of complete DNA-A and DNA-B sequences of BleICV and representative begomoviruses. ML method in MEGA 7 was based on the Tamura-Nei model and the tree support was tested by bootstrapping with 1000 replicates. The scale bar represents the genetic distance. The genomic sequence of *Tomato pseudo-curly top virus* (TPCTV), a topocuvirus, was used as outgrup. For begomovirus acronyms see S1 Table.

Leaves of *Blechum pyramidatum* (Acanthaceae) plants with symptoms of interveinal chlorosis (S1 Fig) were collected in Campeche, Mexico. DNA extracts of dried leaf samples (n = 4) were amplified by polymerase chain reaction (PCR) using the RepDGR-for/ CpYMAC-rev primers. Sequencing of the ~1.4 Kb amplicons from four samples showed that they contained the same virus (sequence identity: >99%). The full DNA-A sequence of the infecting BGV was obtained by sequencing a second amplicon (~1.7 Kb) that overlapped the former PCR product along a ~520 bp segment. The sequence of the virus DNA-B was obtained by an analogous procedure (see Methods). The assembled sequences of genomic components A and B (GenBank accession number: JX827487, JX827488) were compared with those from the NCBI database, and pairwise nucleotide sequence identities were calculated with SDTv1.2. The sequence analyses showed that the closest relative of the Blechum-infecting virus was *Tomato chino La Paz virus* (ToChLPV) at 80% of full DNA-A sequence identity (Fig 1). Based on the current ICTV taxonomic criterion for begomoviruses establishing that a full length DNA-A sequence identity lower than 90% with other BGV is indicative of a separate species [10], and the virus was named *Blechum interveinal chlorosis virus* (BleICV).

The BleICV genome exhibited the typical organization of the New World BGVs. The DNA-A (2645 nt) encoded one open reading frame (ORF) in the virion-sense strand (*AV1*/*CP*), and four ORFs in the complementary strand (*AC1/Rep*, *AC2/TrAP*, *AC3/REn*, and *AC4*). On the other hand, the DNA-B (2640-nt in length) comprised two ORFs: *BV1*/*NSP* in the virion-sense strand and *BC1/MP* in the complementary strand. Both genomic components shared a region of 153 bp displaying a sequence identity of 94.8%; this common region included the stem-loop sequence harboring the invariant nonanucleotide TAATATTAC (the Rep-nicking site), and three GGGGGA iterons (putative Rep-binding sites) with the characteristic arrangement of the NW BGVs (i.e., two repeats in tandem adjacent to the TATA box and one inverted copy closer to the *Rep* gene start codon) [43].

The phylogeny inferred with the ML method implemented in MEGA 7 showed that BleICV is related to viruses belonging to the *Squash leaf curl virus* (SLCV) clade, an ancient lineage of NW BGVs. This relationship is clearest for the DNA-B which is grouped in a distinct branch with three members of the former lineage (i.e., ViYMV, BCaMV and EuMV) (Fig 1B). However, BleICV lacks the distinctive signatures of SLCV clade members, such as number and arrangement of iterons and several amino acid motifs in the N-terminal half of Rep, which are absent in other BGVs.

### Identification of a symmetric phylogenetic footprint in the BleICV *CP* promoter

**Fig 2:**
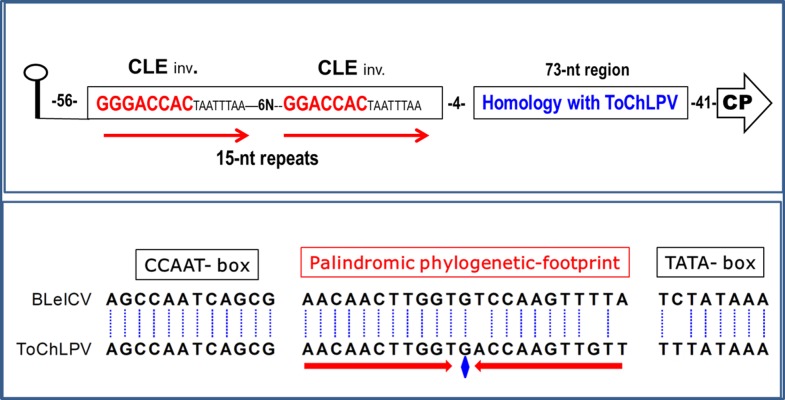
Region of BleICV *CP* promoter encompassing the three phylogenetic footprints (PhyF) shared with ToChLP. *Upper module*. Simplified representation of BleICV *CP* promoter, illustrating the two repeats with canonical CLEs (letters in red). *Lower module*. Phylogenetic footprints in the *CP* promoter of BleICV and ToChLPV (spacing nucleotides between PhyFs were omitted for simplicity). CLE = Conserved Late Element; the arrows denote either direct or inverted repeats; the blue diamond indicates the central nucleotide of the palindromic 23-nt sequence of ToChLPV.

In the course of extensive BLASTn analyses we noted that the non-coding region comprised between the *CP* gene start codon and the conserved stem-loop element of BleICV *Ori*, did not show significant similarity with other begomoviral sequences in public databases, with the sole exception of ToChLPV, with which it shares a 73-bp promoter segment displaying 74% of sequence identity. Visual examination of BleICV sequences upstream of the latter segment identified two direct repeats of 15 bp including a (G)GGACCAC motif, which is the CLE (GTGGTCCC) in inverted orientation. None other begomovirus available in public databases displays analogous repeats, thus explaining the apparent oddness of BleICV *CP* promoter. The 73-bp segment shared with ToChLPV was subsequently analyzed to identify discrete “phylogenetic footprints” (PhyF), which are defined as DNA stretches larger than 6-bp whose sequence, spacing and position relative to other *cis*-acting elements, is conserved in a phylogenetic promoter series of orthologous genes [41, 44]. The evolutionary conservation of a DNA motif in non-coding regions suggests a regulatory function; thus, it is presumed that PhyFs represent binding sites for transcription factors [44]. Three PhyFs were identified in the aforesaid region of BleICV and ToChLPV *CP* promoters: the putative CCAAT and TATA-box elements, and a 23 bp sequence of unknown function located between the latter *cis*-acting elements. In the case of ToChLPV-MM4 (DQ347949) promoter, this PhyF exhibited a perfect dyad symmetry (Fig 2).

### Searching for sequences homologous to the BleICV-ToChLPV symmetric PhyF in New World begomoviruses

**Fig 3:**
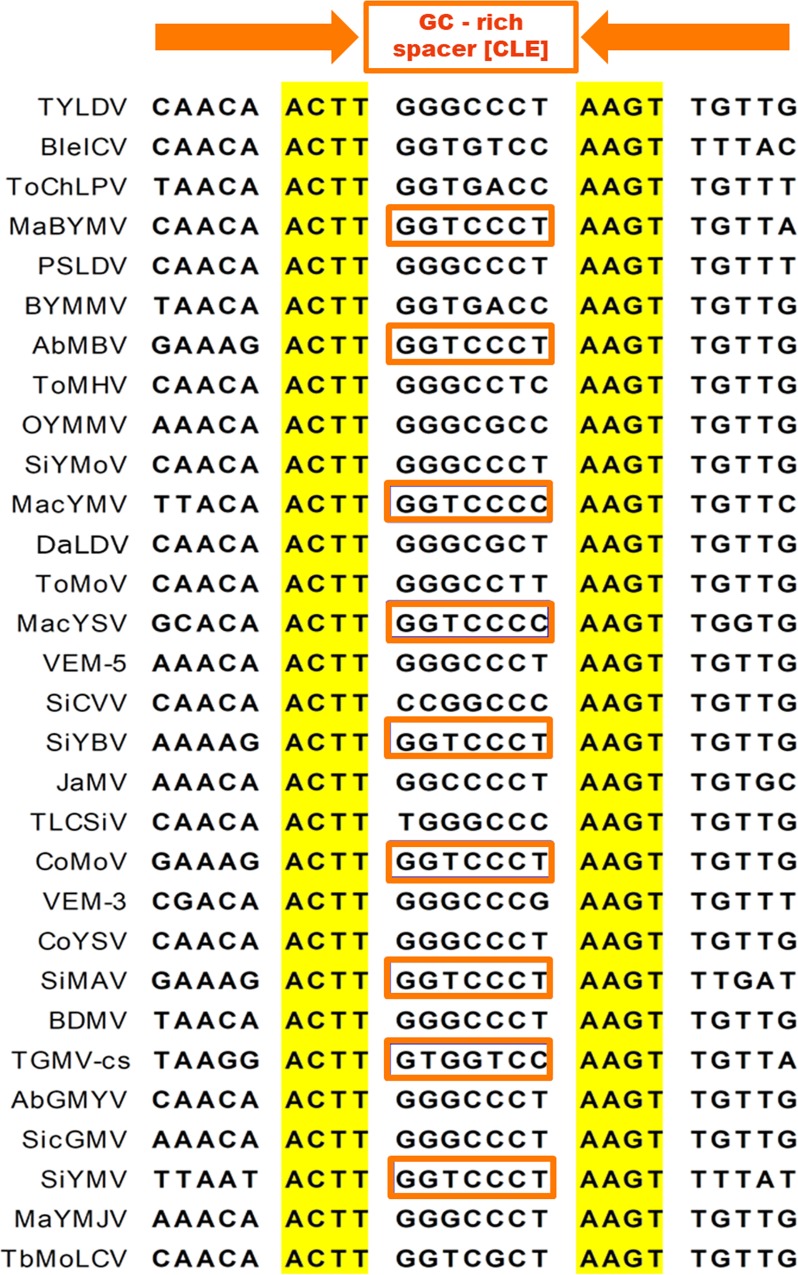
Symmetric region ACTT-(N7)-AAGT of 30 New World begomoviruses. GenBank accesion numbers: TYLDV (KU232891), BleICV (JX827487), ToChLPV (DQ347949), MaBYMV (KU058856), PSLDV (KT899302), BYMMV (FJ944023), AbMBV (JF694480), ToMHV (KT099130), OYMMV (HM035059), SiYMoV (HE806448), MacYMV (AJ344452), DaLCV (JN848773), ToMoV (AY965900), MacYSV (KJ939895), VEM-5 (KT099138), SiCVV (KX691405), SiYBV (KX640991), JaMV (KJ174333), TLCSiV (KY064014), CoMoV (JQ805781), VEM-3 (KT099127), CoYSV (DQ875868), SiMAV (KX896421), BDMV (M88179), TGMV-cs (JF694488), AbGMYV (KC430935), SicGMV (JX857691), SiYMV (AY090558), MaYMJV (FJ601917), TbMoLCV (FM160943).

A BLASTn search for identical or similar sequences to the 23 bp palindromic element of ToChLPV-MM4 produced a single hit at 100% of identity (i.e., *Bean yellow mosaic Mexico virus*, BYMMV), although sequences exhibiting significant but different levels of identity were found in the *CP* promoter of the majority of BGVs native to the Americas. A systematic analysis of those sequences in the ~130 recognized species of New World BGVs was carried out by means of a phylogenetic-structural approach. This analysis revealed short promoter regions that are the evolutionary counterpart to the symmetric BleICV-ToChLPV phylogenetic footprint. Three remarkable common features of the latter regions were discerned: i) they exhibit partial dyad symmetry, with an almost invariant ACTT-N7-AAGT core sequence; ii) the heptanucleotide sequence (N7) between the inverted half-sites is GC-rich and highly variable in sequence; iii) they are invariably associated to the TATA-box element. The sequences of the aforesaid 25 bp-promoter region (hereafter named the “TATA box-associated composite element” or TACE) of 30 New World BGVs are shown in Fig 3. The inferred primeval sequence of this symmetric region, i.e., CAACAACTT-(N7)-AAGTTGTTG, is utterly conserved in a number of BGV species (e.g., in 10 out 30 viruses illustrated in Fig 3), whereas in other BGVs the 9 bp half-sites (“arms”) of the interrupted palindrome are unevenly conserved, as in BleICV and BDMV, that only maintain the original left and right half-sites, respectively. Moreover, in several NW BGVs the dyad symmetry of its TACE is weakly preserved, such as in SiMAV, TGMV and SiYMV (Fig 3).

In contrast to the strong conservation of the TACE core (i.e., ACTT-N7-AAGT), the GC-rich heptanucleotide separating the symmetrical parts of this complex element, exhibited great variability, although some specific sequences occur with higher frequency. For example, among the TACEs illustrated in Fig 3 the spacer sequence was GGGCCCT in nine of them, GGTCCCY in eight, GGTGACC in two, and the remaining eleven TACEs contained distinct spacer sequences. The consensus of the heptanucleotide spacer of the said 30 TACEs was determined (i.e., G28G28K29C24C23C28Y29). Since the spacer is preceded by the double T of the ACTT motif, the TACE 9 bp-central consensus (TTGGKCCC) is remarkably similar to the CLE (GTGGTCCC). To establish the global frequency of heptanucleotide spacers with CLE-like sequences (GGTCCCY or variants differing in a single nucleotide, like GGGCCCY, GGTGCCCY, GGTCGCY, GGTCCTY, etc.) the TACE of the 122 species of NW viruses listed in the review of the genus *Begomovirus* by Brown et al. [10], plus another eight recently described species, were methodically examined. The results of this analysis showed that the variability of the heptanucleotide separating the palindromic half-sites is much greater than that observed in the 30 TACEs illustrated in Fig 3. Nonetheless, a high proportion (~50%) of the examined New World BGVs display TACE with spacers identical or alike to the CLE core. In effect, 15 BGV species exhibit a GGTCCCY motif, whereas 50 viral species display sequences differing on a single nucleotide from the latter motif. Therefore, it can be affirmed that 65 species of NW BGVs have TACEs with "CLE-like" sequences. The names, acronyms, and GenBank accession numbers of those 65 BGVs are summarized in S2 Table.

### Identification of sequences homologous to the TACE in Old World begomoviruses

**Fig 4:**
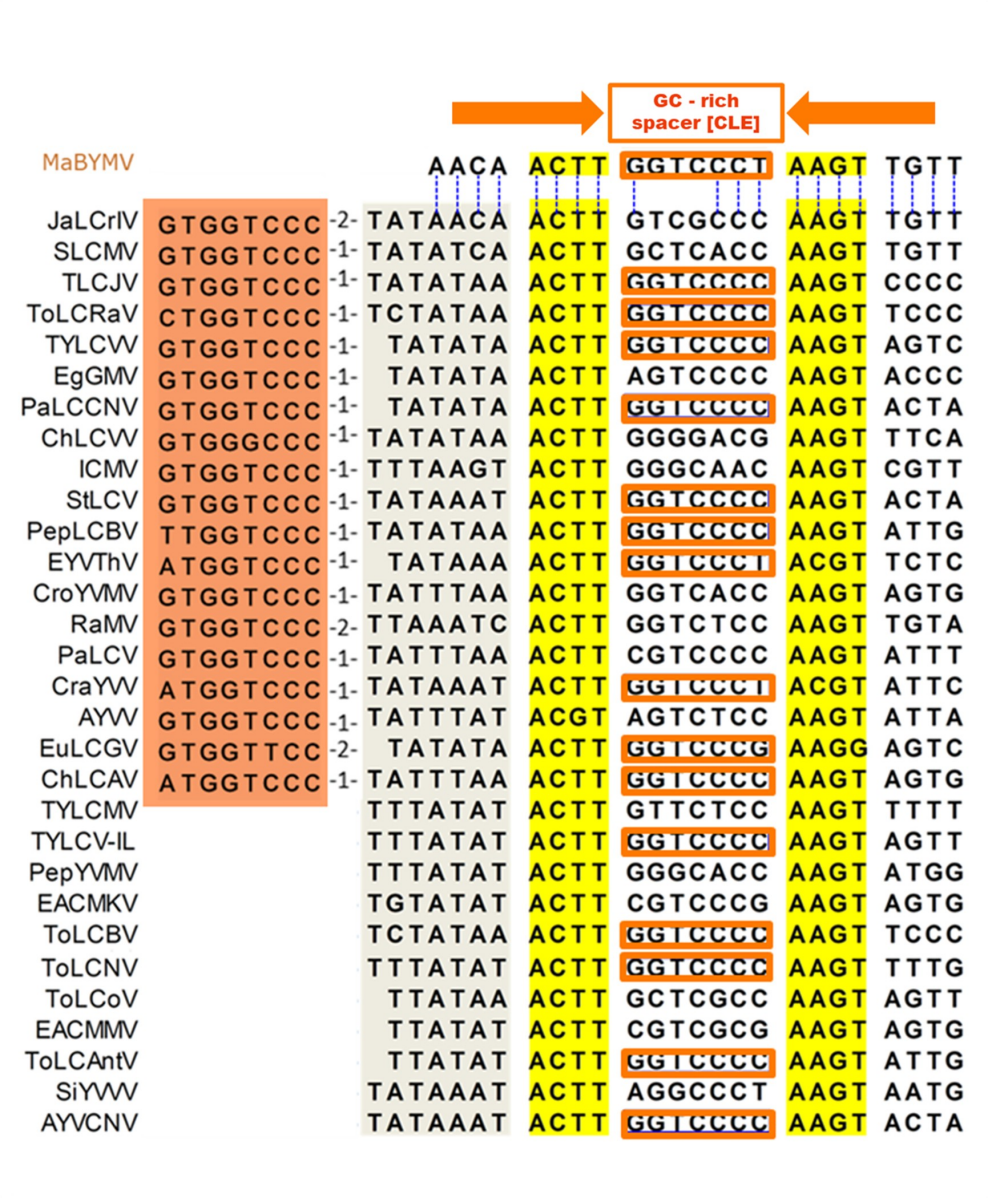
*CP* promoter region containing the TACE of 30 Old World begomoviruses. GenBank accession numbers: MaBYMV (KU058856), JaLCrIV (KM189818), SLCMV (KP455484), TLCJV (KM383747), ToLCRaV (GQ994095), TYLCVV (EU189150), EgGMV (KU569598), PaLCCNV (KU892674), ChLCVV (HM007121), ICMV (Z24758), StLCV (AJ564742), PepLCBV (JN663853), EYVThV (KY373213), CroYVMV (JX270684), RaMV (KX885030), PaLCV (LT009397), CraYVV (FN401520), AYVV (X74516), EuLCGV (AM411424), ChLCAV (KM880103), TYLCMV (LM651401), TYLCV-IL (EF523478), PepYVMV (FM876849), EACMKV (KJ887946), ToLCBV (KM383762), ToLCNV (AM701761), ToLCoV (AJ865341), EACMMV (KP890350), ToLCAntV (AM701767), SiYVVV (KF990601), AYVCNV (HG003652). Note that the first virus, MaBYMV, is an American BGV.

The begomoviruses of continents other than the Americas are collectively called “Old World” BGVs, which are more ancient and diverse than the NW BGVs [45,46]. An important difference between the NW and OW BGVs is that the latter have a small ORF that precedes and partially overlaps the *CP* gene, termed *AV2/V2* or “precoat” gene. Because the NW BGVs presumably evolved of one or more OW lineages [45,46] it was anticipated that the TACE would be present in all or a majority of Old World BGVs. Therefore, it was somehow surprising to find very few sequences of OW BGVs in BLASTn analyses with either the BleICV-ToChLPV phylogenetic footprint or distinct TACEs of other NW BGVs. This unexpected result prompted us to carefully examine the *CP* promoter region of the few Asian BGVs exhibiting a recognizable TACE, such as *Jatropha leaf crumple India virus* (JaCrIV) and *Sri Lankan cassava mosaic virus* (SLCMV). An interesting observation was that the 3´ end of the putative TACE in these viruses is found 10 bp upstream of the *AV2* gene start codon. This position is clearly different from that observed in NW BGVs, in which the TACE is always upstream of the TATA box. Another notable feature of the putative TACEs of JaCrIV and SLCMV is that these are closely associated by its 5'-end with a canonical CLE, an arrangement which was not observed in any NW BGV. Using the TACE of those two Asian viruses as reference, we were able to identify the homologous promoter region in a large number of BGVs native to the Indian subcontinent, Asia, Africa, and Oceania. The relevant features of the OW BGV TACEs are as follows: 1) they contain the same symmetric core ACTT-N7-AAGT like their New World counterparts, but do not exhibit extensive dyad symmetry; 2) the heptanucleotide spacer (N7) sequences are GC-rich and variable in sequence, like its homologous in NW BGVs, but they exhibit more frequently the CLE core sequence; 3) unlike the TACE of NW BGVs, which is consistently upstream of the TATA box, the TACE of the Old World BGVs is downstream of the latter element, which overlaps the TACE left arm; 4) in a considerable number of viruses from the Indian subcontinent and Asia, a canonical CLE is located near the 5 'end of the TACE, separated from it by the putative TATA box. The *CP* promoter region containing the TACE of 30 Old World BGVs is illustrated in Fig 4. An extensive (but not all-inclusive) list of OW BGVs whose heptanucleotide spacer is identical (43 species) or similar (23 species) to the CLE core, is presented in the S2 Table.

### Identification of begomovirus TACE homologous in the *CP* promoter of curtoviruses

Since the CLE is a putative TrAP-responsive element, and a considerable number of begomovirus TACEs include CLE-like spacer sequences and/or are closely associated to CLEs, we were interested to know if geminiviruses of other genera encoding TrAP homologs also contain TACEs in their *CP* promoters. Accordingly, we examined the late promoters of curtoviruses, topocuviruses and turncurtoviruses, looking for sequences homologous to begomovirus TACEs.

**Fig 5:**
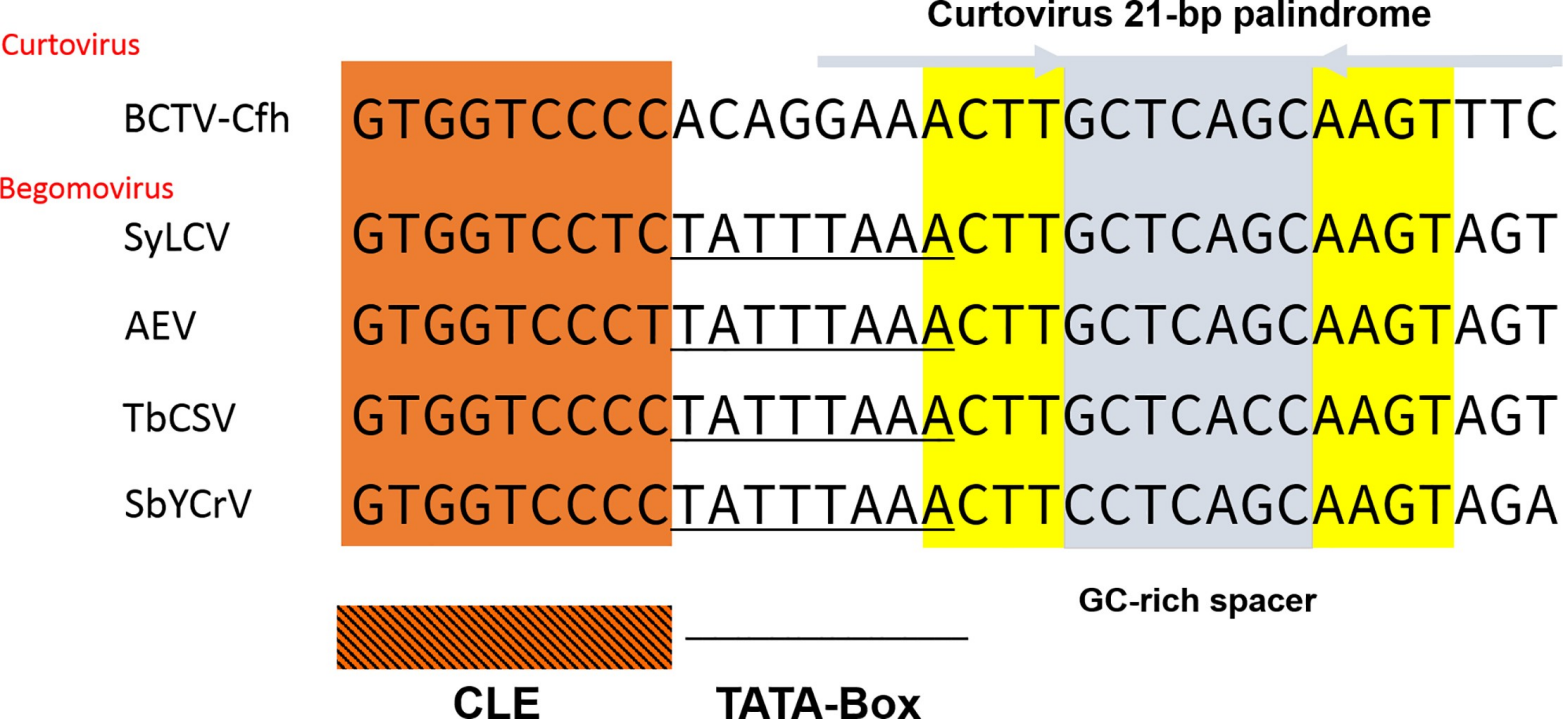
Alignment of the CLE-TACE region of a curtovirus and four Asian begomoviruses. Note the 17 bp segment of the BCTV, SyLCV and AEV TACEs, which is 100% identical in sequence. The distance between the CLE and the TACE is also conserved in these geminiviruses. GenBank accession numbers: BCTV-Cfh (X97203), SyLCV (KU933258), AEV (HE861940), TbCSV (KU934097), SbYCrV (AB050781).

The genus *Curtovirus* includes three species native to North America, but only one of them, *Beet curly top virus* (BCTV), encodes a protein (C2) homologous to TrAP. BCTV comprises several strains that few years ago had the status of separate species. The analysis revealed the existence of BGV TACE homologs in all BCTV strains. For example, in BCTV-Cfh and its relatives the TACE homologous is a palindromic 21 bp sequence displaying 87% of identity with the equivalent element of two Asian BGVs, *Synedrella leaf curl virus* (SyLCV) and *Ageratum enation virus* (AEV). The similarity of the *CP* promoter regions of BCTV-Cfh and the aforementioned BGVs extends upstream of the TACE to include a canonical CLE (Fig 5). The authentic evolutionary relationship of BCTV and begomovirus *CP* promoter regions is further emphasized by its distance from the TATA box, which is similar to that observed in NW BGVs. An additional finding of this analysis was that in several BCTV isolates, such as BCTV-Logan (AF379637), the *CP* promoter contains three copies in tandem of the segment encompassing the CLE-TACE array (not shown) upstream of the TATA-box.

Close examination of the promoter region of the partially overlapped *V3/V2/CP* genes of *Horseradish curly top virus* (HrCTV) [47] and *Spinach severe curly top virus* (SpiSCTV) [48], the two curtoviruses that do not encode TrAP homologs, revealed the existence of TACE homologous in both of them. The HrCTV TACE (5'-AAACTT*GGTCGGC*AAGTTT-3') is a 19-bp sequence partially symmetric, whereas the equivalent element of SpiSCTV is a 25-bp sequence displaying strong dyad symmetry (5'-CAATTCCTT*GCTGGGC*AAGGAATTG-3'), although its central core is CCTT-N7-AAGG, instead the canonical ACTT-N7-AAGT core.

### TACE homologous in CP promoters of topocuvirus and geminiviruses unassigned to a genus

**Fig 6:**
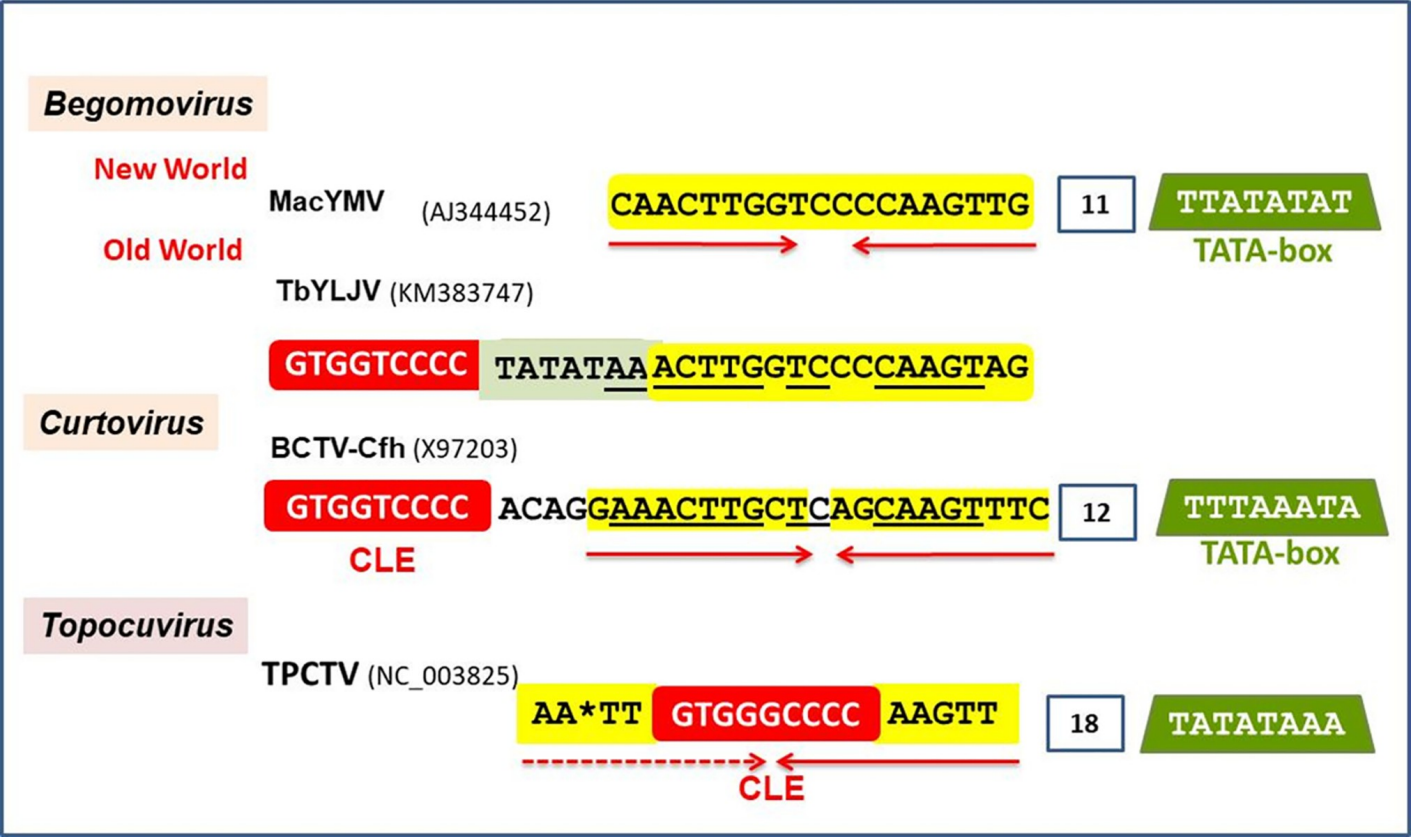
TACE and associated *cis*-acting elements in the *CP* promoter of viruses belonging to three *Geminiviridae* genera. Coloured boxes: red, CLE; yellow, TACE; green, TATA-box. Nucleotides of TbYLJV and BCTV TACEs which are identical are underlined.

Careful scrutiny of the *CP* promoter of the two species of turncurtoviruses currently recognized did not reveal sequences with significant identity to the begomovirus TACE. However, the *CP* promoter of TPCTV, the only topocuvirus described to date, contains an element that by its sequence and proximity to the TATA box can be considered the counterpart of the New World BGV TACE, with a slightly modified 5'-end (Fig 6). The *CP* promoter of viruses belonging to other *Geminiviridae* genera which do not encode TrAP homologs, i.e., *Becurtovirus*, *Capulavirus*, *Eragrovirus*, *Grablovirus* and *Mastrevirus*, were also examined; nonetheless, we were unable to identify homologous to BGV TACEs in the upstream sequences of their late genes.

In addition to the currently recognized genera, there are five species of geminiviruses that have not yet been assigned to a genus due to their peculiar genome organization and high divergence in overall genome sequence. Their characteristics might eventually lead to the establishment of new genera once their insect vector is unequivocally identified. Those geminiviruses include apple [49], grapevine [50], citrus [51], mulberry [52], and tomato [53] pathogens. The analysis of their *CP* promoters revealed, in four out of five geminiviruses, the existence of sequences with the distinctive features of TACEs, that is, a partially symmetric primary structure, a conserved ACTT-N7-AAGT core, a GC-rich heptanucleotide spacer, and nearness to the putative TATA box. The TACEs of these four atypical geminiviruses are illustrated in S2 Fig.

### Synthetic multimers of the CLE fused to a minimal promoter drives high GUS expression in transgenic plants

**Fig 7:**
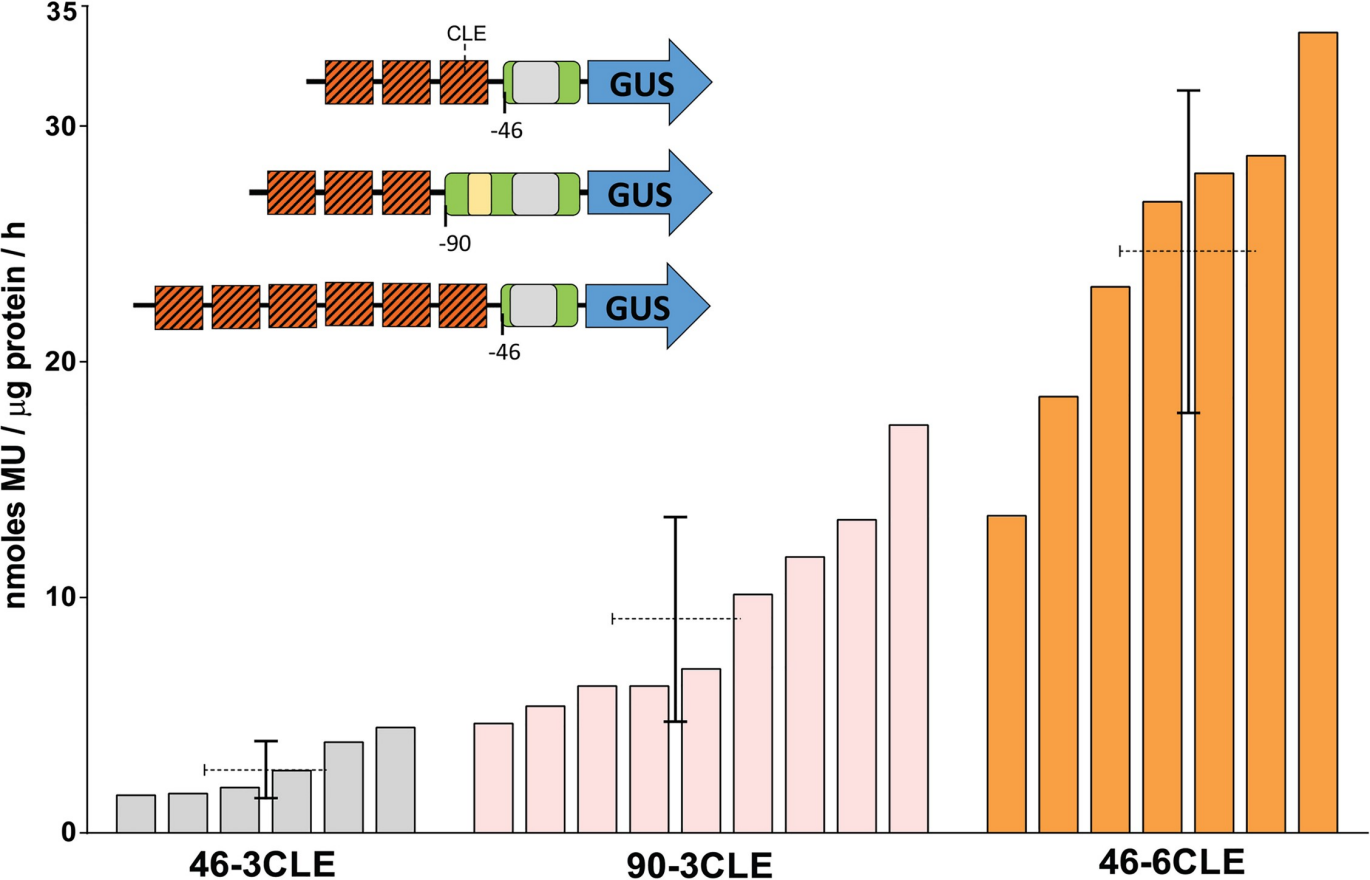
Effect of multiple copies of CLE on GUS expression driven by a truncated -46 and -90 CaMV 35S promoter. Several independent lines of transgenic plants harbouring constructs with synthetic multimers of the CLE upstream of truncated 35S promoters were analysed. Total protein was extracted of the different plant sets and assayed for GUS activity. An average expression (dotted line) and standard deviation are shown for each set of plants. (46-3CLE *n* = 6, 90-3CLE *n* = 9, 46-6CLE *n* = 7).

**Fig 8:**
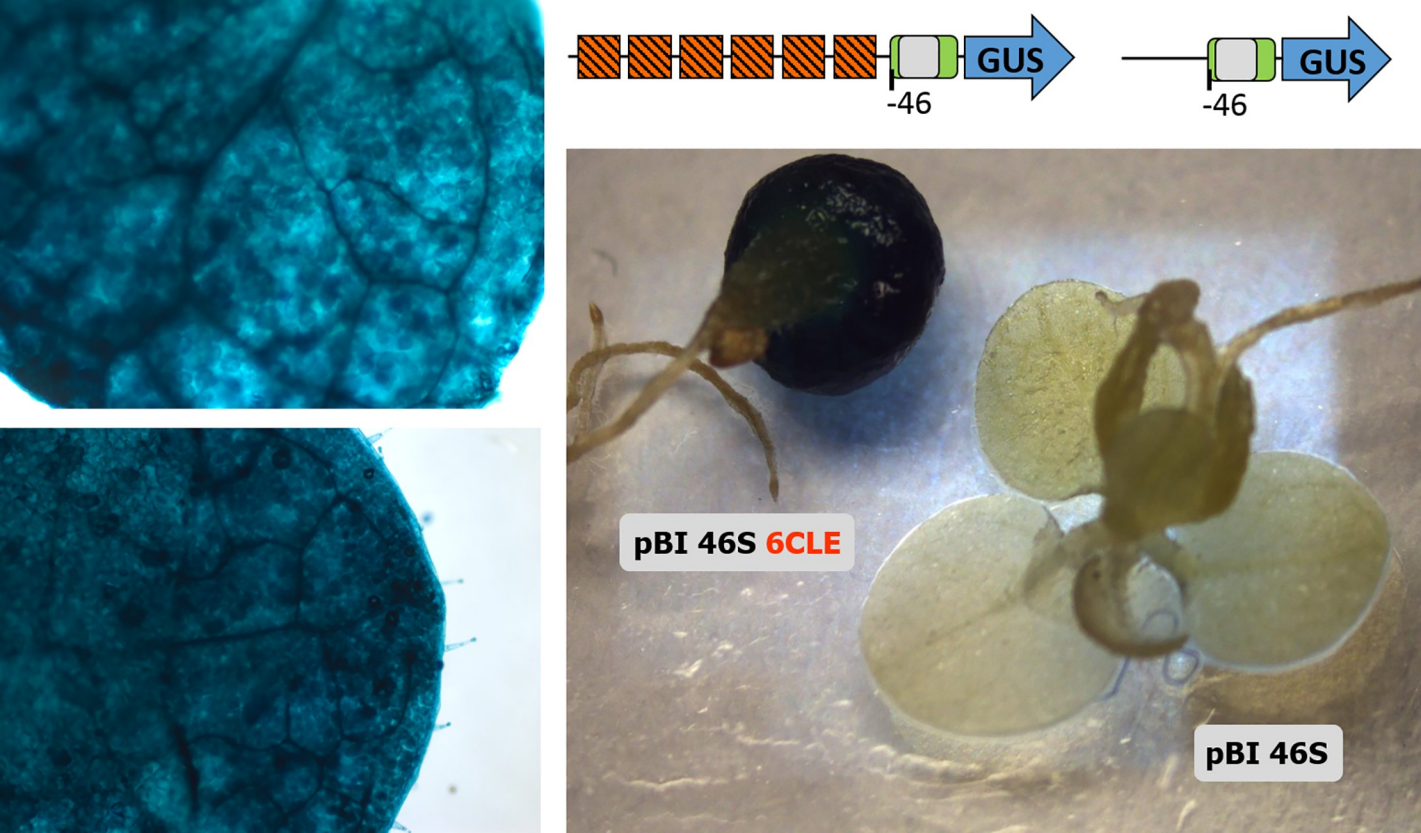
Tissue specific-expression directed by a synthetic promoter containing six copies of the CLE. Transgenic tobacco plantlets harboring a [6CLE-35S prom(-46)-GUS] construct displayed high GUS expression in all leaf cells, including trichomes. A seedling harbouring a [35S prom(-46)-GUS] construct did not express the reporter gene in none of the tissues. The name of the binary vector used to transform the plants is indicated: pBI 46S harbors the [35S prom(-46)-GUS] construct; pBI 46S 6CLE harbors the [6CLE-35S prom(-46)-GUS] construct.

Considering the high proportion of TACEs that exhibit a heptanucleotide spacer identical or similar to the CLE core, as well as the frequent association of the TACE with canonical CLEs, it is really important to clarify the unresolved question of whether this element actually constitutes a functional target of TrAP. In a study conducted in 2005 by Cazonelli et al. [54] it was established that the CLE is recognized by host transcriptional factors, since non-infected transgenic tobacco plants harboring constructs with direct repeats of that element placed upstream from a minimal CaMV 35S promoter-Luc reporter gene cassette, showed luciferase activity in most organs. The level of expression increased directly with the number of CLE copies (4–12). With the aim of establish at a finer level the specific expression pattern directed by CLE multimers, tobacco transgenic plants harbouring chimeric genes [3CLE-35Sprom(-46)-GUS], [3CLE- 35Sprom(-90)-GUS] and [6CLE-35Sprom(-46)-GUS] were generated (see description in Methods). Several independent lines of each of the genetic constructs were obtained, and the beta-glucuronidase activity of F1 seedlings was quantified fluorometrically. As expected, the GUS expression correlated with the number of CLEs: high in plants with the construct harboring 6 copies of the CLE upstream of the minimal CaMV 35S promoter, from high to moderate in plants with 3CLEs upstream of the 35S promoter truncated at position -90, and moderate to low in lines transformed with the construct [3CLE-35S prom(-46)-GUS] (Fig 7). Histochemical staining of transgenic seedlings revealed that the CLE multimers fused to the minimal 35S promoter directed the expression of the reporter gene in the photosynthetic tissues, but not in the stem and root tissues (Fig 8). In lines harboring the construct [6CLE-35S prom(-46)-GUS] the expression of GUS was very strong in all cells with chloroplasts as well as in trichomes (Fig 8).

### Infection of transgenic plants increased the reporter gene expression

**Fig 9:**
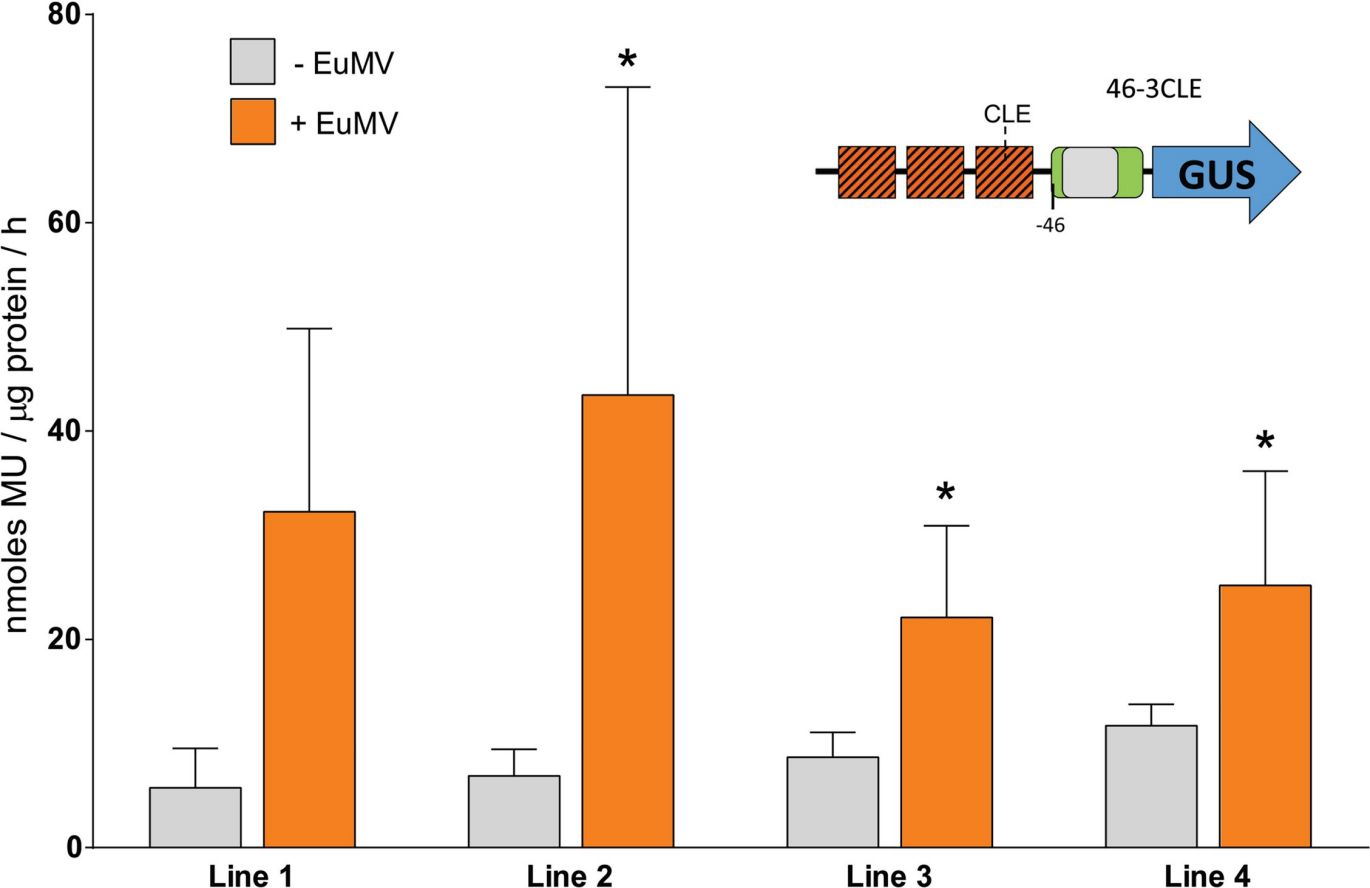
Effect of EuMV infection on GUS expression driven by synthetic promoter containing three copies of the CLE. Four independent transgenic plant lines harbouring the [3CLE-35Sprom(-46)-GUS] construct were tested in the absence or presence of viral factors. Total protein was extracted from the plants 15 dpi and assayed for GUS activity. A two-tailed paired t-test showed GUS expression differences for most lines at *p*<0.05*. Mean and standard deviation are shown. (Line1 *n* = 4, *p* = 0.0869, Line2 *n* = 7, *p* = 0.0125, Line3 *n* = 11, *p* = 0.0010, Line4 *n* = 17, *p* = 0.0002).

As the transgenic plants matured, the GUS activity in the leaves gradually decreased but remained at levels detectable by histochemical techniques in the mesophyll, but not in the leaf veins or in the petiole and stem vascular bundles (not shown). To determine if the transcriptional activity driven by the CLEs multimers was modified in the presence of viral factors, we carried out experiments with four different lines of transgenic plants harboring the [3CLE-35Sprom(-46)-GUS] construct. Plants were inoculated with the bipartite begomovirus *Euphorbia mosaic virus* (EuMV) by particle bombardment. The activity of beta-glucuronidase 15 dpi was quantified and compared with non-inoculated control plants. The expression of the reporter gene in the four transgenic lines was significantly higher in the infected plants than in the uninfected controls (Fig 9). The conclusion derived from these results is that the CLE mediates the increase in transcriptional activity of the promoter and this increment is induced by one or more factors encoded by EuMV, most likely TrAP.

### The mutation and duplication of the CLE in the -125 *CP* promoter of TGMV altered its response to viral factors

**Fig 10:**
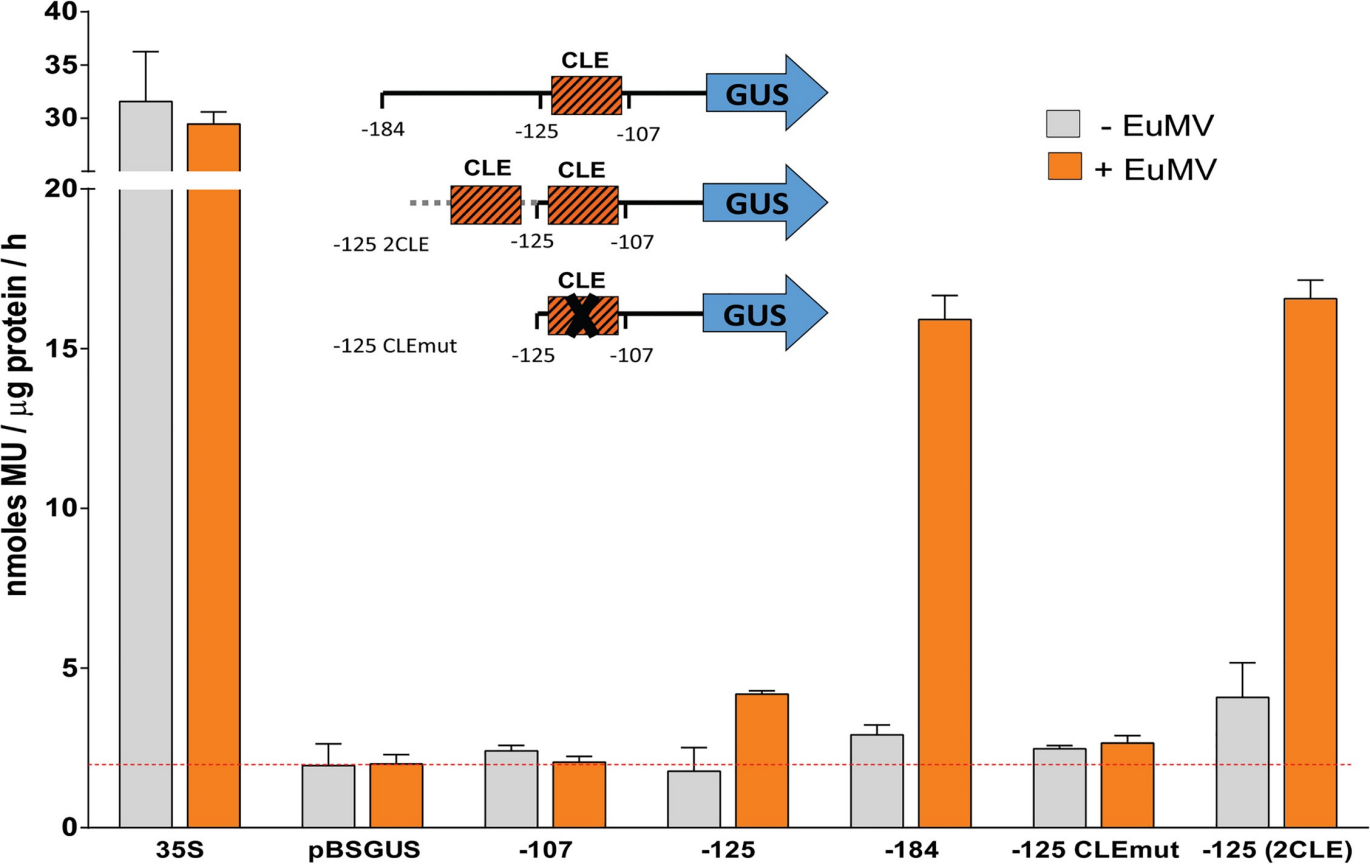
The CLE is a TrAP responsive element. Protoplast transient expression assays were performed with different versions of truncated TGMV *CP* promoter constructs in presence or absence of EuMV DNA-A factors. Total protein of transfected protoplasts was extracted two days post-transformation and assayed for GUS activity. The average and standard deviation of three independent experiments are shown. The red dotted line represents the average expression of empty (promoter-less) vector pBS-GUS in absence of viral factors. This vector was the backbone used for the generation of the different promoter constructs. For comparison purposes a construction with the *uidA* gene driven by the complete CaMV 35S promoter was included in the experiments.

In a pioneering study of the *CP* promoter of TGMV, Sunter and Bisaro [33] concluded that the CLE is not relevant for its activation by TrAP. To determine whether or not this element is critical for the promoter responsiveness to TrAP, we reproduced a series of truncated *CP* promoters examined in the aforementioned study. For this purpose, we designed primers for the PCR amplification of the promoter regions -184/+1, -125/+1, and -107/+1, including the appropriate restriction sites to clone the amplicons into the vector pBS-GUS, which contains the cassette GUS-3'nos derived from pBI121 (see Methods). Additionally, we designed primers to mutate the CLE (changing GTGGTCCC to GTAATAAC) or to add one CLE to the 5´ end of the native -125 promoter, thus producing modified amplicons to generate the constructs -125 CLEmut. and -125 (2CLE), respectively. This collection of truncated *CP* promoters and mutant variants were tested in transient expression assays in tobacco protoplasts, transfecting the constructs individually or in combination with the DNA-A of the begomovirus EuMV, as a source of TrAP. The results of these experiments are shown in Fig 10. As it can be observed, the truncated promoters at positions -184 and -125 significantly increased the expression of the reporter gene in the presence of factors provided by the co-transfected EuMV DNA-A, while the promoter truncated at -107 did not show responsiveness to viral factors. These results are analogous to those obtained by Sunter and Bisaro [33]. The most interesting results were obtained with the constructs -125 CLEmut and -125 (2CLE). Indeed, the CLE mutation abolished the transcriptional response of the -125 promoter to proteins expressed by EuMV-A, hence indicating that the CLE is the element mediating the transactivation of the promoter by viral factors. This conclusion was corroborated with the response of the -125 (2CLE) promoter, that increased the expression of GUS to a similar level to that of the truncated -184 promoter. Interestingly enough, the -125 (2CLE) promoter showed higher basal activity than the -125 promoter, an observation that is consistent with the additive effect of CLE copies on the promoter basal activity reported by Cazonelli et al. [54]. Consequently, the ratio between the transcriptional activity of the -125 (2CLE) promoter in the presence and absence of viral factors is lower than that determined for the -184 promoter (S3 Fig).

## Discussion

In this study, the complete genome of a novel bipartite begomovirus native to Southeast Mexico, *Blechum interveinal chlorosis virus*, was characterized. Comparative analyses of its DNA-A intergenic region revealed a 23-bp long PhyF with dyad symmetry in the proximal region of the *CP* promoter. A search for this PhyF homologs in New World BGVs unveiled a collection of related sequences that are composed by two discernible kind of elements. Because of its complex composition and consistent association with the TATA-box we dubbed it “TATA-associated composite element” (TACE). The equivalent element in the *CP* promoter of Old World BGVs has a different position relative to the TATA-box, which is placed upstream of the TACE and partially overlaps its left arm (Figs 4 and 5). Begomovirus TACE homologs were also identified in members of other two *Geminiviridae* genera, and four atypical geminiviruses currently unassigned to a genus.

Several questions naturally arise from the present study findings: What is the TACE function? Why does the heptanucleotide spacer varies in sequence, not only between viral species, but even between isolates of the same species? Which host and/or virus factors interact with that complex element? Searches in databases specialized in *cis*-regulatory elements and transcriptional factors of plants, such as PlantCare [55], PLACE [56], and PlantPAN [57] did not provide significant data to ascertain the probable function of the palindromic arms of New World BGV TACEs (i.e., CAACAACTT-(N7)-AAGTTGTTG). In the case of the GC-rich spacer sequence, our data indicate that it is related to the CLE, without being identical or even similar to that element in a considerable number of BGVs. Indeed, a comprehensive analysis showed that in (at least) 66 species of Old World BGVs the TACE spacer is similar to the CLE core, whereas ~50% of the New World BGVs exhibit CLE-like spacer sequences in their TACEs. Furthermore, canonic CLEs are closely associated with the TACE in a significant number of viruses native to the Indian subcontinent and Asia (Figs 4 and 5). This association of the TACE with CLE-like sequences is particularly clear in some divergent lineages of BGVs, such as the New World SLCV clade, the Asian lineage of *Tomato leaf curl New Delhi virus* (ToLCNDV), and the lineage of the so-called “sweepoviruses”, probably originated in China.

The *CP* promoters of BGVs belonging to the SLCV lineage (~30 species) commonly contain two or three copies of the CLE (in direct or inverse orientation) one of which is placed between the TACE and the TATA box (S4 Fig). A single member of this lineage (i.e., *Cabbage leaf curl virus*) exhibits an incomplete TACE but retains the CLE adjacent to the TATA box (S4 Fig). On the other hand, eleven BGVs related to ToLCNDV display a CLE adjacent to the TATA box, like many other OW BGVs, but that CLE is in reverse orientation (S5 Fig). Finally, the 13 recognized species of sweepoviruses, that have the smallest *CP* promoters among all BGVs (i.e., 89 to 126 bp in length), exhibit a very short TACE which is only composed of the ACTT (N7) AAGT core; this element is not associated with the TATA box, but it is immediately adjacent to the *V2* gene, a unique position observed among geminiviruses. The heptanucleotide spacer of sweepovirus TACEs differs in one or two nucleotides of the CLE core. Moreover, the *CP* promoter of all sweepoviruses contains a canonical CLE downstream the conserved stem-loop element, and three species exhibit a second CLE in reverse orientation, which together with the first CLE forms a long interrupted palindrome (S6 Fig).

### The CLE is an element of response to TrAP

As mentioned in the introductory section, the experimental evidence obtained by different laboratories has been contradictory. So, for example, Ruiz-Medrano et al. [32] that studied the PHYVV *CP* promoter, reported experimental data consistent with the CLE hypothesis, whereas Sunter and Bisaro [33] concluded that this conserved element is not involved in the TrAP-mediated activation of TGMV *CP* promoter, because the deletion of the single CLE present in it did not affect its TrAP-responsiveness (in the context of the entire intergenic region). In the present study, we have shown that the CLE is really necessary for the transactivation of the TGMV *CP* promoter truncated at position -125. What is more, the artificial addition of one CLE upstream of the -125 *CP* promoter significantly enhanced its transactivation by TrAP. A plausible explanation of the results obtained by Sunter and Bisaro in their study of 2003 [33] is that there is one, or more, TrAP responsive element different to the CLE upstream of the -125 nucleotide of TGMV *CP* promoter. This alternative interpretation is consistent with data from the same study, which showed that the level of transactivation of the truncated promoter at position -184 practically doubled the -125 promoter activity.

The comparative analysis of the smallest truncated *CP* promoters that have shown responsiveness to TrAP, like -115 PHYVV [32], -125 TGMV [33] and -151 CbLCV [24] promoters, also led to the conclusion that the CLE is plausibly the *cis*-acting element mediating the response to the transactivator, because those truncated promoters only have an element in common besides the TATA-box, i.e., the CLE. An important corollary of the validation of the CLE as a TrAP-responsive element is that this opens new prospects to identify plant genes functioning as primary targets of the transactivator. Indeed, TrAP is a key factor of begomoviruses ability to reprogram the host to backing viral infection and to evade plant defense responses, as suggested by the observation that the expression of the AC2 gene of ACMV and CbLCV strongly altered the transcriptomes of tobacco and Arabidopsis, respectively [58, 59]. It is very plausible that the promoters of some of the host genes responding directly to the presence of TrAP contain CLEs, and therefore could be identified by bioinformatics approaches in the genome of model plants. Recently, Babu et al [60] undertook a genome-wide mapping of CLEs in *Arabidopsis thaliana* and identified 122 promoters containing exact GTGGTCCC motifs. Interestingly enough, several of the identified promoters correspond to genes encoding transcription factors of the MYB, WRKY and BHLH DNA binding families, thus suggesting the indirect regulation by TrAP of multiple plant genes involved in plant antiviral defenses and enhanced support of virus infection [60].

### What regulatory function does the TACE have?

In this study, it was showed that transgenic tobacco plants harboring synthetic promoters with either 3 or 6 copies of the CLE upstream of the 35S minimal promoter expressed moderate (i.e., 3CLEs) or strong (6CLEs) beta-glucuronidase activity in mesophyll cells and trichomes, but not in mature vascular tissues (Figs 7 and 8). It has been established that TrAP interacts with diverse DNA-binding proteins, including PEAPOD2 (PPD2) of Arabidopsis [30]. Purified PPD2 bound to the so-called *CP* promoter activator region of TGMV (-166 to -59) in electrophoretic mobility shift assays (EMSA), but the PPD2 binding site could not be identified with precision. Recently, Gonzalez et al. [61] carried out a genome-wide determination of PPD2 target sites, and they found among 2042 peak sequences identified, that two specific motifs were highly represented. The first motif, present in 726 peak sequences, was GmCACGTGkC. The second motif was yctCACGCGCyt, which was present in 275 peak sequences. The PPD2 factor is a repressor, that downregulates the expression of its target genes [61]. So, it is possible that PPD2 could be involved in the repression of *CP* and other late promoters in the absence of TrAP. Indeed, is currently no clear understanding of the molecular mechanisms involved in the transcriptional processes mediated by TrAP; however, the consistent association of the TACE with the TATA box of *CP* promoters, in addition to the similarity of the heptanucleotide spacer with either the CLE or the PPD2 target sequences, suggests that this composite element could be involved in the TrAP-mediated derepression of the *CP* gene in the phloem.

## Supporting information

S1 Table Names, acronyms and GenBank accession numbers of geminiviruses compared in Fig 1. (PDF)

S2 Table Begomoviruses exhibiting TACE spacer sequences identical or similar to the CLE core. (PDF)

S3 Table Oligonucleotides used in this study. (PDF)

S4 Table Experimental data of GUS activity presented in Figs 7, 9 and 10, and S3 Fig. (XLSX)

S1 Fig Symptoms caused by BleICV in *Blechum pyramidatum* plants. (PDF)

S2 Fig TACE and associated putative *cis*-acting elements in four geminiviruses unassigned to a genus. (PDF)

S3 Fig Expression of different CLE-containing *CP* promoters in presence of EuMV factors. (PDF)

S4 Fig *CP* promoter region with the TACE-CLE-TATA box conserved arrangement in members of the SLCV lineage. (PDF)

S5 Fig *CP* promoter region containing the inverted CLE-TATA box-TACE arrangement in members of the ToLCNDV lineage. (PDF)

S6 Fig Complete V2/CP promoter of selected Sweepoviruses, a divergent lineage of Old World begomoviruses. (PDF)
