# Supplementary material for: Analysis of a new begomovirus unveils a composite element conserved in the CP gene promoters of several Geminiviridae genera: Clues to comprehend the complex regulation of late genes
Source: PLoS One. 2019 Jan 23;14(1):e0210485. doi: 10.1371/journal.pone.0210485 (PMC6344024; doi:10.1371/journal.pone.0210485)
Supplement: S1 Table — (PDF) [file pone.0210485.s001.pdf]

Analysis of a new begomovirus unveils a composite element conserved in the *CP* gene promoters of several *Geminiviridae* genera: clues to comprehend the complex regulation of late genes.

Mariana Cantú-Iris1, Jorge Armando Mauricio-Castillo 2, Guillermo Pastor-Palacios3, Bernardo Bañuelos-Hernández4, Jesús Aarón Avalos-Calleros1, Alejandro Juárez-Reyes, Rafael Rivera-Bustamante, Gerardo Rafael Argüello-Astorga.1*

Supporting information- **S1 Table**

Legend

Names, acronyms and GenBank accession numbers of geminiviruses compared in S1 Fig.

S1 Table. Names, acronyms and GenBank accession numbers of geminiviruses compared in S1 Figure.

Virus	Acronym	GenBank Accession	
		DNA-A	DNA-B
Blechum interveinal chlorosis virus	**BleICV**	**JX827487.1**	**JX827488.1**
Tomato yellow leaf curl virus	TYLCV-IL	X15656.1	------
Tomato golden mosaic virus-yv	TGMV	K02029.1	K02030.1
Chino del tomate virus-IC	CdTV-IC	AF101476.1	AF101478.1
Bean calico mosaic virus	BCaMV	AF110189.1	AF110190.1
Cabbage leaf curl virus	CaLCuV	U65529.2	U65530.2
Pepper huasteco yellow vein virus-Sinaloa	PhYVV	AY044162.1	AY044163.1
Macroptilium mosaic Puerto Rico virus	MaMPRV	AY044133.1	AF449193.1
Corchorus yellow vein Vietnam virus	CoYVV	AY727903.1	AY727904.1
Tomato yellow spot virus	ToYSV	DQ336350.1	DQ336351.1
Euphorbia mosaic virus-Jalisco	EuMV-B	DQ520942.1	HQ185235.1
Sida mosaic Sinaloa virus	SiMSiV	DQ520944.1	DQ356428.1
Corchorus golden mosaic virus	CoGMV	DQ641688.1	DQ641689.1
Clerodendrum golden mosaic China virus	ClGMCNV	FJ011668.1	FJ011669.1
Sida mosaic Bolivia virus	SiMBoV1	HM585441.1	NC_015044.1
Tomato severe leaf curl virus-Huasteca	ToSLCV-HU	JN680352.1	------
Tomato chino La Paz virus	ToChLPV-SLP	JN676150.1	------
Datura leaf distortion virus	DaLDV	JN848773.1	JN848774.1
Tomato yellow margin leaf curl virus	TYMLCV	AY508993.2	AY508994.2
Vigna yellow mosaic virus	VYMV	KC430936.1	KC430937.1
Common bean mottle virus	CBMoV	KX011473.1	KX011474.1

Papaya leaf curl Guandong virus-W4	PaLCuGuV-W4	KF446660.1	------
Papaya leaf curl Guandong virus-W1	PaLCuGuV-W1	KF446659.1	------
Tomato pseudo-curly top virus	TPCTV	X84735.1	------
Bean dwarf mosaic virus	BDMV	M88179.1	M88180.1
Macroptilium yellow mosaic virus-Cuba	MacYMV	AJ344452.1	------
Tomato mottle virus	ToMoV	AY965900.1	AY965901.1
Tomato chino La Paz virus isolate Solanum MM4	ToChLPV	DQ347949.1	------
Corchorus yellow spot virus	CoYSV	DQ875868.1	DQ875869.1
Bean yellow mosaic Mexico virus	BYMMV	FJ944023.1	------
Okra yellow mosaic Mexico virus	OYMMV	HM035059.1	HM035060.1
Abutilon mosaic Brazil virus	AbMBV	JF694480.1	JF694479.1
Tomato golden mosaic virus isolate csTGMV	TGMV-cs	JF694488.1	JF694489.1
Corchorus mottle virus	CoMoV	JQ805781.1	JQ805780
Abutilon golden mosaic Yucatan virus	AbGMYV	KC430935.1	------
Sida yellow mottle virus	SiYMoV	HE806448.1	------
Jatropha mosaic virus	JaMV	KJ174333.1	KJ174337.1
Macroptilium yellow spot virus	MacYSV	KJ939895.1	------
VEM begomovirus 3 isolate GtSq10	VEM-3	KT099127.1	------
Tomato mosaic Havana virus	ToMHV	KT099130.1	KT099164.1
VEM begomovirus 5 isolate PR4-7	VEM-5	KT099138.1	------
Passionfruit leaf distortion virus	PSLDV	KT899302.1	NC_031765.1
Tomato yellow leaf distortion virus	TYLDV	KU232891.1	KU232892.1
Sida chlorotic vein virus	SiCVV	KX691405.1	KX691413.1
Malvastrum bright yellow mosaic virus	MaBYMV	KU058856.1	KU058860.1

Sida mottle Alagoas virus	SiMAV	KX896421.1	------
Sida yellow blotch virus	SiYBV	KX640991.1	KX640992.1
Tomato leaf curl Sinaloa virus	TLCSiV	KY064014.1	
