# Supplementary material for: Analysis of a new begomovirus unveils a composite element conserved in the CP gene promoters of several Geminiviridae genera: Clues to comprehend the complex regulation of late genes
Source: PLoS One. 2019 Jan 23;14(1):e0210485. doi: 10.1371/journal.pone.0210485 (PMC6344024; doi:10.1371/journal.pone.0210485)
Supplement: S2 Table — (PDF) [file pone.0210485.s002.pdf]

Analysis of a new begomovirus unveils a composite element conserved in the *CP* gene promoters of several *Geminiviridae* genera: clues to comprehend the complex regulation of late genes.

Mariana Cantú-Iris[1], Jorge Armando Mauricio-Castillo [2], Guillermo Pastor-Palacios[3], Bernardo Bañuelos-Hernández[4], Jesús Aarón Avalos-Calleros[1], Alejandro Juárez-Reyes, Rafael Rivera-Bustamante, Gerardo Rafael Argüello-Astorga.[1*]

Supporting information- **S2 Table**

Names, acronyms and GenBank accession numbers of begomoviruses exhibiting TACE spacer sequences identical or similar to the CLE core.

S2 Table - Begomoviruses exhibiting TACE spacer sequences identical or similar to the CLE core.

Old World begomoviruses<br><br>**ACTT GGTCCCY AAGT**		
Virus	Acronym	Gen Bank Accesion
Ageratum yellow vein China virus	AYVCNV	KU954379
Ageratum leaf curl virus-[G52]	ALCuV	AJ851005
Ageratum yellow vein virus	AYVV	JN809811
Alternanthera yellow vein virus	AlYVV	KX710155
Cotton leaf curl Gezira virus	CLCuGeV	HG969203
Chayote yellow mosaic virus	ChaYMV	KT454827
Chilli leaf curl Salem virus	ChiLCSV	HM007119
Chilli leaf curl virus	ChiLCV	HM992939
Chilli leaf curl Ahmedabad virus	ChiLCAV	KM880103
Chilli leaf curl Multan virus	ChiLCMV	JN663865
Chili leaf curl Pakistan virus	ChiLCPKV	HM587709
Chilli leaf curl Kanpur virus	ChiLCKV	HM007106
Emilia sonchifolia yellow vein Thailand virus	EYVThV	KY373213
Emilia yellow vein virus	EYVV	KJ016240
Erectites yellow mosaic virus	ErYMV	DQ641698
Hollyhock leaf crumple virus	HoLCV	AJ542539
Lycianthes yellow mosaic virus	LyYMV	KT582302
Malvastrum leaf curl Guangdong virus	MLCuGdV	KJ016236
Malvastrum leaf curl virus	MaLCuV	EF554783
Mimosa yellow leaf curl virus	MiYLCV	DQ641695
Pepper leaf curl Bangladesh virus	PepLCBV	AF314531
Pepper leaf curl virus	PepLCV	AF414287
Stachytarpheta leaf curl virus-[Hn5]	StaLCuV	AJ495814
Telfairia mosaic virus	TeMV	KU683743
Tobacco curly shoot virus	TbCSV	JN387045
Tobacco leaf curl Comoros virus	TbLCKMV	AM701760
Tomato leaf curl Anjouan virus	ToLCAnV	AM701758
Tomato leaf curl Bangladesh virus	ToLCBV	KM383765
Tomato leaf curl Guangdong virus	ToLCGdV	AY602165
Tomato leaf curl Guangxi virus	ToLCGxV	KJ524905
Tomato leaf curl Hainan virus	ToLCHaiV	FN256261
Tomato leaf curl Hanoi virus	ToLCHaV	HQ162270
Tomato leaf curl Joydebpur virus	ToLCJV	JQ654463

Tomato leaf curl Namakely virus	ToLCNV	AM701761
Tomato leaf curl Sulawesi virus	ToLCSuV	FJ237620
Tomato leaf curl Sudan virus	ToLCSdV	KT760555
Tomato leaf curl Ranchi virus	ToLCRnV	JN676053
Tomato yellow leaf curl Thailand virus	TYLCTHV	KX290922
Tomato yellow leaf curl virus	TYLCV	KJ585666
Tomato yellow leaf curl Guangdong virus	TYLCGdV	GQ169042
Tomato yellow leaf curl Mali virus	TYLCMLV	DQ358913
Tomato yellow leaf curl Vietnam virus	TYLCVV	EU189150
Vernonia yellow vein Fujian virus	VeYVFV	JF265670
**A single mismatch-GGTCCCY core**		
*Cotton leaf curl Gezira virus**	CLCuGeV	FN554523
Chilli Leaf curl Vellanad virus	ChiLCVV	HM007121
Desmodium mottle virus	DesMoV	KY294725
Pepper leaf curl Lahore virus	PepLCLaV	JN663864
Papaya leaf curl virus	PaLCuV	LN845913
Pepper yellow leaf curl Thailand virus	PepYLCThV	KT322145
Pepper yellow vein Mali virus	PepYVMLV	AM691547
Ramie mosaic virus	RamMV	KC171650
Soybean chlorotic blotch virus	SbCBV	KT444614
Squash leaf curl Yunnan virus	SLCuYV	KX388157
Sweet potato leaf curl China virus	SPLCCNV	DQ512731
Sweet potato leaf curl Georgia virus	SPLCGV	AF326775
Sweet potato leaf curl Henan virus	SPLCHnV	KC907406
Sweet potato leaf curl Uganda virus	SPLCUV	FR751068
Sweet potato leaf curl virus	SPLCV	EU856364
Sweet potato mosaic virus	SPMV	FJ969831
Tomato leaf curl Cotabato virus	ToLCCoV	EU487048
Tomato leaf curl Gandhinagar virus	ToLCGaV	KC952005
Tomato leaf curl Gujarat virus	ToLCGuV	LN794215
Tomato leaf curl Karnataka virus	ToLCKaV	KP178731
Tomato leaf curl virus	ToLCV	JX416176
Tomato leaf curl Patna virus	ToLCPatV	KM383745
Tomato yellow leaf curl China virus	TYLCCNV	KU934105
**New World begomoviruses**<br>**ACTT GGTCCCY AAGT**		
Abutilon mosaic Brazil virus	AbMBV	JF694481
Corchorus mottle virus	CoMoV	JQ805781
Leonurus mosaic virus	LeMV	JX863082
Malvastrum bright yellow mosaic virus	MaBYMV	KU058871
Macroptilium yellow mosaic virus	MacYMV	EF582840
*Macroptilium yellow spot virus**	MacYSV	KJ939887

Macroptilium yellow vein virus	MacYVV	KJ939908
Pepper huasteco yellow vein virus	PHYVV	LN848886
*Sida micrantha mosaic virus**	SiMMV	FN557522
Sida mottle Alagoas virus	SiMAV	KX896427
Sida mottle virus	SiMoV	JX871378
Sida yellow blotch virus	SiYBV	KX640991
Sida yellow mosaic virus	SiYMV	AY090558
Tomato yellow margin leaf curl virus	ToYMLCV	AY508993
Tomato yellow spot virus	ToYSV	KX348176
**A single mismatch-GGTCCCY core**		
*Abutilon mosaic Brazil virus**	AbMBV	FN4344380
Bean chlorosis virus	BChV	JN848770
Bean golden mosaic virus	BGMV	KJ939849
Bean yellow mosaic Mexico virus	BYMMV	FJ944023
Chenopodium leaf curl virus	ChLCV	HM626515
Common bean severe mosaic virus	CBSMV	KX011476
Corchorus yellow spot virus	CoYSV	DQ875868
Cnidoscolus mosaic leaf deformation virus	CnMLDV	KT966771
Jacquemontia yellow mosaic virus	JacYMV	KF661331
Jacquemontia yellow vein virus	JacYVV	KY624376
Jatropha mosaic virus	JaMV	KJ174333
Macroptilium bright mosaic virus	MacBMV	KX691399
Macroptilium common mosaic virus	MacCMV	KX691397
Macroptilium yellow mosaic Florida virus	MacYMFV	AY044135
*Macroptilium yellow spot virus**	MacYSV	KJ939897
Malvastrum bright yellow mosaic virus	MaBYMV	KU058862
Malvastrum yellow mosaic Helshire virus	MaYMHeV	FJ600483
Malvastrum yellow mosaic Jamaica virus	MaYMJV	FJ601917
Okra mottle virus	OMoV	EU914817
Oxalis yellow vein virus	OxYVV	KM887907
Passionfruit leaf distortion virus	PasLDV	KT899302
Pepper golden mosaic virus	PepGMV	LN848789
Rhynchosia golden mosaic Yucatan virus	RhGMYuV	KT381193
*Sida micrantha mosaic virus**	SiMMV	KX691410
Sida mosaic Sinaloa virus	SiMSiV	DQ520944
Sida yellow mottle virus	SiYMoV	JN411687
Sida ciliaris golden mosaic virus	SicGMV	JX857691
Sida golden mosaic Brazil virus	SiGMBRV	FN436001
Sida golden mosaic Florida virus-[A1]	SiGMFlV	U77963
Sida golden mosaic Lara virus	SiGMLaV	JX857693
Sida golden mosaic virus	SiGMV	GQ357649
Sida golden mosaic Braco virus	SiGMBcV	JX162595
Sida golden yellow vein virus 1	SiGYVV	AJ577395

Sida golden yellow vein virus 2	SiGYVV	KT879828
Sida yellow mosaic Yucatan virus	SiYMYuV	DQ875872
Sida yellow net virus	SiYNV	KU996355
Soybean chlorotic spot virus	SbChSV	KJ939918
Tobacco leaf rugose virus	TbLRV	AJ488768
Tobacco mottle leaf curl virus	TbMoLCV	FM160943
Tobacco yellow crinkle virus	TbYCV	KU562964
Tomato chino La Paz virus	ToChLPV	DQ347948
Tomato chlorotic leaf distortion virus	ToClLDV	HQ201952
Tomato chlorotic mottle Guyane virus	ToCMoGV	KR263181
Tomato mottle leaf curl virus	ToMoLCV	KY196218
Tomato mottle leaf curl virus	ToMoLCV	KX896411
Tomato severe leaf curl virus	ToSLCV	JN680352
Tomato yellow leaf distortion virus	ToYLDV	KU232891
Tomato wrinkled mosaic virus	ToWMV	KY449275
Triumfetta yellow mosaic virus	TrYMV	KU131588
VEM begomovirus 4	VEM-4	KT099128
VEM begomovirus 5	VEM-5	KT099142
Wissadula golden mosaic St Thomas Virus	WGMV	DQ395343
