# Supplementary material for: Analysis of a new begomovirus unveils a composite element conserved in the CP gene promoters of several Geminiviridae genera: Clues to comprehend the complex regulation of late genes
Source: PLoS One. 2019 Jan 23;14(1):e0210485. doi: 10.1371/journal.pone.0210485 (PMC6344024; doi:10.1371/journal.pone.0210485)
Supplement: S3 Table — (PDF) [file pone.0210485.s003.pdf]

Analysis of a new begomovirus unveils a composite element conserved in the *CP* gene promoters of several *Geminiviridae* genera: clues to comprehend the complex regulation of late genes.

Mariana Cantú-Iris1, Jorge Armando Mauricio-Castillo 2, Guillermo Pastor-Palacios3, Bernardo Bañuelos-Hernández4, Jesús Aarón Avalos-Calleros1, Alejandro Juárez-Reyes, Rafael Rivera-Bustamante, Gerardo Rafael Argüello-Astorga.1*

Supporting information- **S3 Table**

Oligonucleotides used in this work.

Table S3

**Oligonucleotide**	**Sequence 5'- 3'**
A-3CLE	AGCTTGACTA**GTGGTCCC**CGCTAGCA**GTGGTCCC**AAGGCCTA**GTGGTCCC**GTTAACT
A-3CLEcomp	CTAGAGTTAACGGGACCACTAGGCCTTGGGCAAACTGCTAGCGGGGACCACTAGTCA
B-3CLE	AGCTTAAGTG**GTGGTCCC**CCAAGATCTA**GTGGTCCC**CATATGA**GTGGTCCC**AGTACTT
B-3CLEcomp	AGCTAAGTACTGGGACCACTCATATGGGGACCACTAGATCTTGGGGACCACTTA
TGMV -107	ATAAAGCTTGCATATGTGAAGGGCCAAT
TGMV -125	ATAAAGCTTTCGTCTAA**GTGGTCCC**GC
TGMV -184	ATAAAGCTTACCGGATGGCCGCGCGAT
TGMV 125-2CLE	ATAAGCTT**GTGGTCCC**TCGTCTAA**GTGGTCCC**G
TGMV CLEmut	ATAAAGCTTTAA**GTAATAAC**GCATATGTGAAGG
