# Supplementary material for: Analysis of a new begomovirus unveils a composite element conserved in the CP gene promoters of several Geminiviridae genera: Clues to comprehend the complex regulation of late genes
Source: PLoS One. 2019 Jan 23;14(1):e0210485. doi: 10.1371/journal.pone.0210485 (PMC6344024; doi:10.1371/journal.pone.0210485)
Supplement: S2 Fig — (PDF) [file pone.0210485.s006.pdf]

Analysis of a new begomovirus unveils a composite element conserved in the *CP* gene promoters of several *Geminiviridae* genera: clues to comprehend the complex regulation of late genes.

Mariana Cantú-Iris[1], Jorge Armando Mauricio-Castillo [2], Guillermo Pastor-Palacios[3], Bernardo Bañuelos-Hernández[4], Jesús Aarón Avalos-Calleros[1], Alejandro Juárez-Reyes, Rafael Rivera-Bustamante, Gerardo Rafael Argüello-Astorga.[1*]

Supporting information- **S2 Fig**

S2 Figure.

TACE and associated putative *cis*-acting elements in four geminiviruses unassigned to a genus. Coloured boxes: red, CLE; yellow, TACE arms; gray, TACE spacer sequence; gree, TATA-box. The start codon of the first ORF in the virion-strand sense is indicated.

Geminivirus species unassigned to a genus

Apple geminivirus **(AGmV)** (KM386645)

GTGGGCCCC -34- ACAACTTCCCCTGCAAGTT-8-TTTTATTA --49-- ATG

Grapevine geminivirus A (GGVA) (KX570611)

GTGGTCCC-22-CTACTACTTGGGGCCTAAGTTATTATA --26-- ATG

Mulberry mosaic dwarf associated virus (MMDaV) (KP699129)

GTGGGTCCCATGAATAATTATTAAAGAGGTTGCTTGCGCAGCAAGGAACCGATG

Tomato apical leaf curl virus (ToALCV) (MG491196)

CTATTACTTGCTTCGCAAGTAATCTTATTATAA ---27-- ATG
