# Supplementary material for: Analysis of a new begomovirus unveils a composite element conserved in the CP gene promoters of several Geminiviridae genera: Clues to comprehend the complex regulation of late genes
Source: PLoS One. 2019 Jan 23;14(1):e0210485. doi: 10.1371/journal.pone.0210485 (PMC6344024; doi:10.1371/journal.pone.0210485)
Supplement: S3 Fig — (PDF) [file pone.0210485.s007.pdf]

Analysis of a new begomovirus unveils a composite element conserved in the *CP* gene promoters of several *Geminiviridae* genera: clues to comprehend the complex regulation of late genes.

Mariana Cantú-Iris[1], Jorge Armando Mauricio-Castillo [2], Guillermo Pastor-Palacios[3], Bernardo Bañuelos-Hernández[4], Jesus Aaron Avalos-Calleros[1], Alejandro Juárez-Reyes, Rafael Rivera-Bustamante, Gerardo Rafael Argüello-Astorga.[1*]

Supporting information- S3 Figure

S3 Figure.

Expression of different CLE containing CP promoters in presence of EuMV factors. Transient expression assays were performed on protoplasts in absence or presence of viral factors. Each bar represents mean of fold activation (Ratio [TrAP-mediated / Basal] GUS activity) for each promoter construct set of three experiments. Standard deviations are shown. The -125, -184, -125(2CLE) constructs were statistically significant at $p<0.001$ in relation to TrAP non responding vector pBSGUS (red dotted line) or promoter -107 construct lacking CLE element.

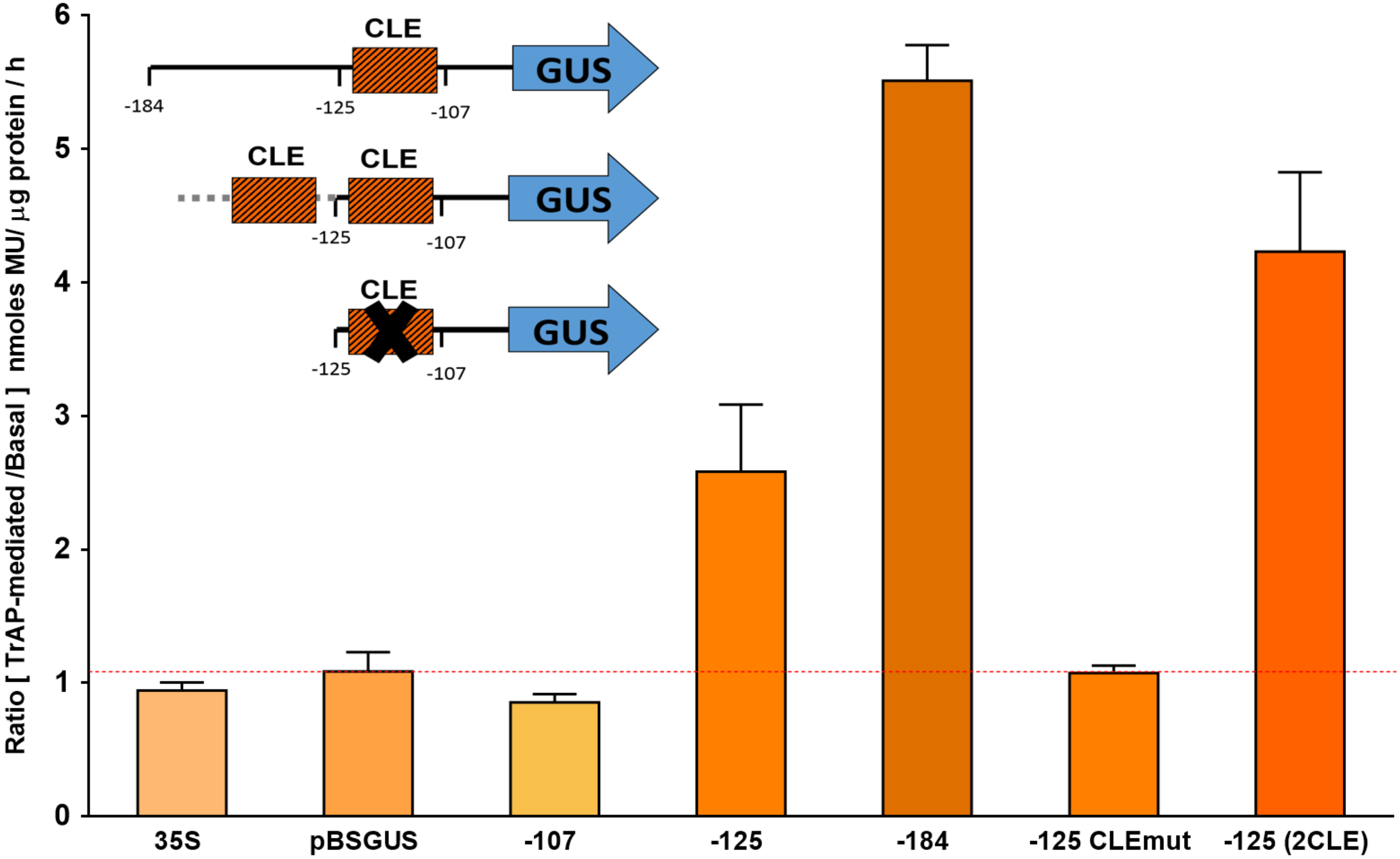

Ratio [TrAP-mediated /Basal] nmoles MU/ μg protein / h
0
1
2
3
4
5
6
CLE
GUS
-184
-125
-107
CLE
CLE
GUS
-125
-107
CLE
GUS
-125
-107
35S
pBSGUS
-107
-125
-184
-125 CLEmut
-125 (2CLE)
