# Supplementary material for: Analysis of a new begomovirus unveils a composite element conserved in the CP gene promoters of several Geminiviridae genera: Clues to comprehend the complex regulation of late genes
Source: PLoS One. 2019 Jan 23;14(1):e0210485. doi: 10.1371/journal.pone.0210485 (PMC6344024; doi:10.1371/journal.pone.0210485)
Supplement: S4 Fig — (PDF) [file pone.0210485.s008.pdf]

Analysis of a new begomovirus unveils a composite element conserved in the *CP* gene promoters of several *Geminiviridae* genera: clues to comprehend the complex regulation of late genes.

Mariana Cantú-Iris[1], Jorge Armando Mauricio-Castillo [2], Guillermo Pastor-Palacios[3], Bernardo Bañuelos-Hernández[4], Jesús Aarón Avalos-Calleros[1], Alejandro Juárez-Reyes, Rafael Rivera-Bustamante, Gerardo Rafael Argüello-Astorga.[1*]

Supporting information- **S4 Fig**

***CP* promoter region with the TACE-CLE-TATA box conserved arrangement in members of the SLCV lineage.** Coloured boxes: red, CLE; yellow, TACE arms; gray, TACE spacer; green, TATA-box

BGVs of the SLCV lineage

CP promoter region encompassing the TACE-TATA box array.

Squash leaf curl virus (DQ285016)

AATGCCTTGGTGACTAAGTGTGGTCCCTATAAAAAAG

Squash mild leaf curl virus (NC_004645)

TATAGCTTGGTGCATAAGTGTGGTCCCTATAAATTAA

Melon chlorotic leaf curl virus (AF325497)

TATGCCTTGGTGAGTCAGCGTGGTCCCTATAAATGA

Squash yellow mild mottle virus (KC153490)

AATGACTTGGTGACTAAGCGTGGTCCCTATAAATTAA

Cabbage leaf curl virus (U65529)

GGCTAACTGAGTGCGCTGTGGGCCTATATAAAGA

Rhynchosia golden mosaic Yucatan virus (KT381193)

TTTTGACTTGGTCACCAAGTTGTGGGCCTATATAACGT

Euphorbia yellow mosaic virus (KY559452)

AGTCTACTTGGCCGATAAGTTGTGGTCCCTATATATTTA

Euphorbia mosaic Peru virus (AM886131)

GTCCTACTTGCTGAGTAAGTTGTGGTCCCTATATTAATA

Euphorbia mosaic virus (DQ520942)

TCATGACTTACCCACCAAGCTGTGGTCCCTATAAATAAA

Cucurbit leaf curl virus (AF224760)

TATGCTTTCTTGACTAAGACGTGGTCCTTTATCTATAAA

Tomato mild yellow leaf curl Aragua virus (KY353797)

TTTAACTTATTCGTTAAGTTGTGGTCCCTATATATTTA

Bean leaf crumple virus (KX857725)

TTAAGACTTAGCGCCTAAGTTGTGGGCCTATAAATAAA

Pepper golden mosaic virus (GU128148)

TTGTGCCTTGGTGACCAAGGTGTGGTCCTATAAAT

Rhynchosia mosaic Sinaloa virus (DQ406672)

TTGTGACTTGGCGACCAAGTTGTGGGCCTATAAAACG

Abutilon golden mosaic Yucatan virus (KC430935)

CAACAACTTGGGCCCTAAGTTGTTGGTCCGTTATAAATTAA

Capraria yellow spot Yucatan virus (KC426927)

TTATTACTTGCTGACTAAGTGTGGTCCCTGCCTATTAAAG

Coloured boxes: red, CLE; yellow, TACE arms; gray, TACE spacer; green, TATA-box
