# Supplementary material for: Analysis of a new begomovirus unveils a composite element conserved in the CP gene promoters of several Geminiviridae genera: Clues to comprehend the complex regulation of late genes
Source: PLoS One. 2019 Jan 23;14(1):e0210485. doi: 10.1371/journal.pone.0210485 (PMC6344024; doi:10.1371/journal.pone.0210485)
Supplement: S5 Fig — (PDF) [file pone.0210485.s009.pdf]

Analysis of a new begomovirus unveils a composite element conserved in the *CP* gene promoters of several *Geminiviridae* genera: clues to comprehend the complex regulation of late genes.

Mariana Cantú-Iris[1], Jorge Armando Mauricio-Castillo [2], Guillermo Pastor-Palacios[3], Bernardo Bañuelos-Hernández[4], Jesus Aaron Avalos-Calleros[1], Alejandro Juárez-Reyes, Rafael Rivera-Bustamante, Gerardo Rafael Argüello-Astorga.[1*]

Supporting information- S5 Figure

Legend.

***CP* promoter region containing an inverted CLE- TATA box-TACE arrangement in members of the ToLCNDV lineage.** Coloured boxes: red, CLE; green, putative TATA-box; yellow, TACE arms; gray, TACE spacer. The start codon of the precoat (V2) gene is underlined.

BGVs related to *Tomato leaf curl New Delhi virus* (ToLCNDV)

(Inverted CLE associated to the TACE)

Tomato leaf curl New Delhi virus AM258977

GGGGACCAATAAATAGACTTGCTCACCAAGTTTGGATCCACAAACATG

Tomato leaf curl Palampur virus KY564204

GGGGACCAATAAATAGACTTGCTCACCAAGCTTGGATCCACAAACATG

Loofa yellow mosaic virus F509739.

GGGGACCATTAAATAGACTTCCTCACCAAGTTTGGATTCAAAACATG

Bitter gourd yellow vein virus KM190927

GGGGACCAATAAATAGACTTGCTCACCAAGTCTTCATACACCAACATG

Chilli leaf curl Bijnour virus KC465466

GGGGACCAATAAATAGACTTGCTCACCAAGTTTGGATCCACCAACATG

Squash leaf curl China virus MF102264

GGGGACCAATAAATAGACTTGCTCACCAAGTTTGGATCTACAAACATG

Squash leaf curl Philippines virus EU487033

GGGGACCATTAAATAGACTTCCGCACCAAGTTTTGATCCACAACATG

Pumpkin yellow mosaic Malaysia virus EF197941

GGGGACCATTAAATAGACTTCCTCACCAAGTTTTGATCCACACCATG

Chayote enation yellow mosaic virus KX259339

GGGGACCAATAAATAGAATTGCTCAACAAGTTCGGATCCACAAACATG

Melon leaf curl virus AM494976

GGGGACCAATAAATAGACTTGCTCACCAAGCTTGGATCCACAAACATG

Papaya leaf crumple virus KX302711

GGGGACCAATAAATAGACTTGCTCACCAAGTTTGTATCTACAAACATG
