# Supplementary material for: Analysis of a new begomovirus unveils a composite element conserved in the CP gene promoters of several Geminiviridae genera: Clues to comprehend the complex regulation of late genes
Source: PLoS One. 2019 Jan 23;14(1):e0210485. doi: 10.1371/journal.pone.0210485 (PMC6344024; doi:10.1371/journal.pone.0210485)
Supplement: S6 Fig — (PDF) [file pone.0210485.s010.pdf]

Analysis of a new begomovirus unveils a composite element conserved in the *CP* gene promoters of several *Geminiviridae* genera: clues to comprehend the complex regulation of late genes.

Mariana Cantú-Iris[1], Jorge Armando Mauricio-Castillo [2], Guillermo Pastor-Palacios[3], Bernardo Bañuelos-Hernández[4], Jesús Aarón Avalos-Calleros[1], Alejandro Juárez-Reyes, Rafael Rivera-Bustamante, Gerardo Rafael Argüello-Astorga.[1*]

Supporting Information- **S6 Fig**

Legend.

Complete V2/CP promoter of selected Sweepoviruses, a divergent lineage of Old World begomoviruses. Coloured boxes: red, CLE; green, putative TATA-box; yellow, TACE arms; gray, TACE spacer. The start codon of the precoat (V2) gene is indicated with black bold letters.

Complete *V2/CP* promoter of Sweepoviruses

(The stem-loop element of viral *Ori* is omitted)

Sweet potato leaf curl China virus DQ512731

GCCTCCCTTTTAAT**GTGGACCCC**ACACGCTTTACTTTAATTATTAAAGACGTGCCTGTT
GCAGACTTGGTCGCCAAGA**ATG**

Sweet potato leaf curl Georgia virus AF326775

CGCGCCCTTTTAAA**GTGGGCCCC**ACACGCTTTGCTTTAATTATTTAAAGGCGTGCCTGG
TGTAGACTTGGTCGCCAAGT**ATG**

Sweet potato leaf curl Sao Paulo virus HQ393477

GTTCGTTTTATG**GTGGGCCCC**ACACGGCATCTTTAATCATTAAAAGGCGCGTCAATTAC
GAGACTTTGTCGCCAAGT**ATG**

Sweet potato leaf curl Uganda virus FR751068

CGCGCCCTTTAATT**GTGGGCCCC**ACACGCTTTACTTTAATCATTAAAAGGCGTGCCTGT
TGCAGTCTTGGTCGCCAAGA**ATG**

Sweet potato leaf curl virus- [Spain isolate] EU856364

CGCGCCCTTTTAAA**GTGGGCCCC**ACATT**GGGGACCAC**GCGTCTTTTCTGTTCTTACTTT
AATGATGACATTGCTTTATAAGGACCAATGCTGTTCCAGTCTTGGTGCCCAAGT**ATG**

Sweet potato leaf curl Guangxi virus KJ476510

GCGCCTCTTTTTTT**GTGGGCCCC**ACAA**GGGGACCAC**GCGCCTTTACTTTAATGCTTTAA
TGATGACTCCACTTTAAATTGGCCAATCCTGTTCCAGTCTTGGTCGCCAAGG**ATG**
